# Integrative analysis with machine learning identifies diagnostic and prognostic signatures in neuroblastoma based on differentially DNA methylated enhancers between INSS stage 4 and 4S neuroblastoma

**DOI:** 10.1007/s00432-024-05650-4

**Published:** 2024-03-21

**Authors:** Shan Li, Tao Mi, Liming Jin, Yimeng Liu, Zhaoxia Zhang, Jinkui Wang, Xin Wu, Chunnian Ren, Zhaoying Wang, Xiangpan Kong, Jiayan Liu, Junyi Luo, Dawei He

**Affiliations:** 1 Department of Urology, Children’s Hospital of Chongqing Medical University, Zhongshan 2nd Road, No. 136, Yuzhong District, Chongqing, 400014 China; 2 Chongqing Key Laboratory of Children Urogenital Development and Tissue Engineering, Chongqing, 400014 China; 3 China International Science and Technology Cooperation Base of Child Development and Critical Disorders, National Clinical Research Center for Child Health and Disorders, Ministry of Education Key Laboratory of Child Development and Disorders, Chongqing Key Laboratory of Pediatrics, Children’s Hospital of Chongqing Medical University, Chongqing, 400014 China

**Keywords:** Neuroblastoma, Machine learning, Prognostic prediction, DNA methylation, Single-cell analysis

## Abstract

**Introduction:**

Accumulating evidence demonstrates that aberrant methylation of enhancers is crucial in gene expression profiles across several cancers. However, the latent effect of differently expressed enhancers between INSS stage 4S and 4 neuroblastoma (NB) remains elusive.

**Methods:**

We utilized the transcriptome and methylation data of stage 4S and 4 NB patients to perform Enhancer Linking by Methylation/Expression Relationships (ELMER) analysis, discovering a differently expressed motif within 67 enhancers between stage 4S and 4 NB. Harnessing the 67 motif genes, we established the INSS stage related signature (ISRS) by amalgamating 12 and 10 distinct machine learning (ML) algorithms across 113 and 101 ML combinations to precisely diagnose stage 4 NB among all NB patients and to predict the prognosis of NB patients. Based on risk scores calculated by prognostic ISRS, patients were categorized into high and low-risk groups according to median risk score. We conducted comprehensive comparisons between two risk groups, in terms of clinical applications, immune microenvironment, somatic mutations, immunotherapy, chemotherapy and single-cell analysis. Ultimately, we empirically validated the differential expressions of two ISRS model genes, CAMTA2 and FOXD1, through immunochemistry staining.

**Results:**

Through leave-one-out cross-validation, in both feature selection and model construction, we selected the random forest algorithm to diagnose stage 4 NB, and Enet algorithm to develop prognostic ISRS, due to their highest average C-index across five NB cohorts. After validations, the ISRS demonstrated a stable predictive capability, outperforming the previously published NB signatures and several clinic variables. We stratified NB patients into high and low-risk group based on median risk score, which showed the low-risk group with a superior survival outcome, an abundant immune infiltration, a decreased mutation landscape, and an enhanced sensitivity to immunotherapy. Single-cell analysis between two risk groups reveals biologically cellular variations underlying ISRS. Finally, we verified the significantly higher protein levels of CAMTA2 and FOXD1 in stage 4S NB, as well as their protective prognosis value in NB.

**Conclusion:**

Based on multi-omics data and ML algorithms, we successfully developed the ISRS to enable accurate diagnosis and prognostic stratification in NB, which shed light on molecular mechanisms of spontaneous regression and clinical utilization of ISRS.

**Supplementary Information:**

The online version contains supplementary material available at 10.1007/s00432-024-05650-4.

## Introduction

Neuroblastoma (NB), one of the most common pediatric extracranial solid malignancies, presents unique challenges and opportunities for innovative diagnostic and therapeutic strategies (Tsubota and Kadomatsu 2018). Researchers frequently utilized the International Neuroblastoma Staging System (INSS) to stratify patients with NB, and defined INSS stage 4S as NB patients aged less than one year, having a localized primary tumor with limited metastasis to the liver, skin, or bone marrow (tumor cells less than 10%) (Brodeur et al. 1993). Despite patients with stage 4 and 4S shared similar clinical features, stage 4 patients owned significantly worse prognoses than stage 4S patients (Ikeda et al. 2002), while spontaneous regression of NB is commonly observed in stage 4S patients (Papac 1996).

The heterogeneity of NB, particularly highlighted in the differences between INSS stage 4 and 4S NB, necessitates a comprehensive understanding of its molecular underpinnings to guide effective treatments (Brodeur 2018). Nevertheless, the specific response mechanism underlying the spontaneous regression of NB remains elusive (Papac 1998), while extensive efforts have been worked on this process based on mechanisms such as autophagy (Meng et al. 2020; Inoue et al. 2009) and apoptosis (Koizumi et al. 1995; Kocak et al. 2013). This phenomenon has not been fully explained by transcriptomics alone, prompting investigations into the DNA methylome of stage 4S NB. Research has shown that certain chromosomal regions are particularly rich in promoters with differential methylation specific to stage 4S. Additionally, these studies have identified a distinct pattern of hypermethylation in specific subtelomeric promoters. These findings provide a deeper understanding of the biological processes underlying the unique tumor biology of stage 4S, as well as its tendency for spontaneous regression (Decock et al. 2016).

Recent advancements in bio-informatics technologies have ushered in a new era of NB therapy. The integration of targeted panel sequencing offers a robust and scalable method for analyzing a wide array of genomic NB risk markers in a single assay (Szymansky et al. 2021). The comprehensive sequencing of NB cell lines has elucidated differential expression patterns based on various genetic aberrations or phenotypes, such as MYCN amplification status and ALK mutation status (Harenza et al. 2017). DNA methylation studies have pinpointed specific genes, such as PHGDH, related to serine metabolism, which are strongly expressed and characteristically methylated in certain aggressive NB subgroups (Watanabe et al. 2022). Meanwhile, single-cell transcriptomics has been instrumental in identifying chemoresistance-associated genes and pathways and understanding intra-tumor heterogeneity (ITH) in high-risk NB cases (Avitabile et al. 2022).

In conclusion, the utilization of transcriptomic profiling, methylation analysis, and single-cell RNA sequencing in NB research could revolutionize our understanding of the complex distinctions between INSS 4 and 4S NB. With the assistance of machine learning algorithms (Liu et al. 2022), we aimed to explore an innovative approach for diagnosing INSS 4 NB patients, as well as appraising the effectiveness of immunotherapy and forecasting the prognosis of NB patients, based on a large amount of multi-omics data.

## Materials and methods

### Datasets

**Fig. 1:**
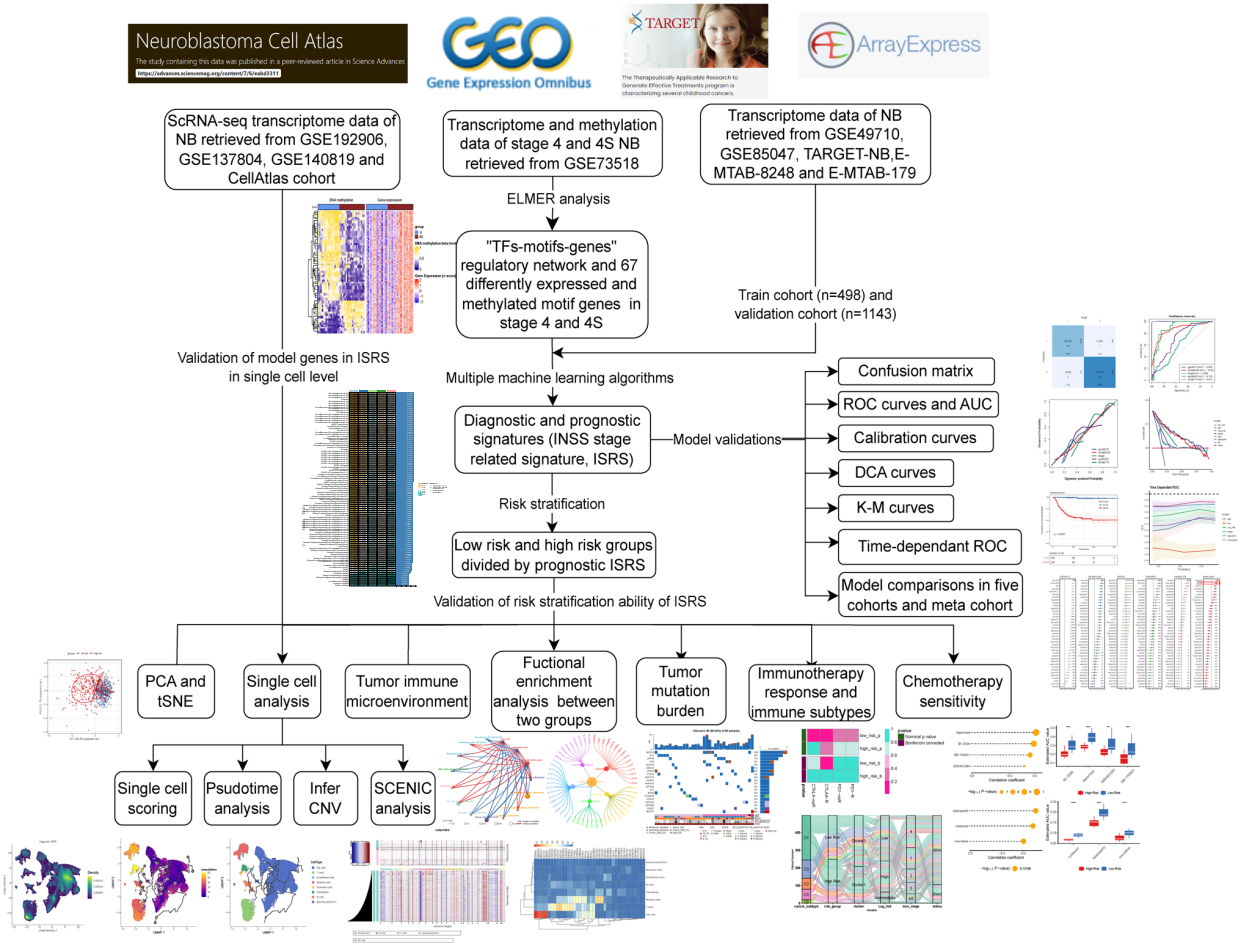
General workflow of our bioinformatics study

Ten cohorts were utilized in our study. Dataset GSE73518 containing transcriptome and methylation data of 105 NB patients was obtained from the GEO database. We utilized the champ.norm function of “ChAMP” R package to normalize the methylation data. Moreover, we obtained five independent transcriptome datasets including GSE49710 and GSE85047 cohorts from the GEO database, TARGET-NB cohort from the TARGET database, and E-MTAB-8248 and E-MTAB-179 cohorts from the ArrayExpress database. We removed patients with incomplete follow-up data and enrolled 1617 patients for the following analysis. We utilized the GSE49710 cohort to be the training cohort in which the prognostic and diagnostic models were established. Meanwhile, the other four datasets were set as the validation cohort to verify the stability of the models. Detail information on NB patients in bluk-seq cohorts was provided in Supplementary Table S1. The transcriptomic data was normalized with log2 (*x* + 1) algorithm and the combat function of “sva” R package was utilized to correct batch effects (Leek et al. 2012). Besides, three scRNA-seq datasets involving 31 patients with INSS 4 and 4S NB patients were obtained with accession number GSE192906, GSE137804, GSE140819 from GEO database. Another scRNA-seq cohort was downloaded from https://www.neuroblastomacellatlas.org/, which contains scRNA-seq data published in a peer-reviewed article (namely CellAtlas dateset in the following analysis) (Kildisiute et al. 2021). Detail information on NB patients in four scRNA-seq cohorts was listed in Supplementary Table S2. The bioinformatics data used in this study is sourced from open-access platforms and is publicly available, thus negating the need for ethical approval or patient consent. The workflow diagram of our analysis is provided in Fig. 1.

### Enhancer-associated regulatory network

Using the “ELMER” R package, we analyzed gene methylation and transcription data from NB samples in the GSE73518 cohort, focusing on the sequences of differentially methylated probes between INSS stage 4 and 4S NB. (Silva et al. 2019). Enhancer Linking by Methylation/Expression Relationships (ELMER) analysis revealed enriched motifs and predicted the transcription factors (TFs) interacting with these motifs, leading to the construction of a "TFs-motifs-genes" regulatory network. ELMER analysis involved five key steps: (1) Identifying distal probes (those more than 2 kb upstream from the transcription start site) in the methylation chip data; (2) detecting variations in methylation levels between normal and tumor samples; (3) determining target genes for the differentially methylated probes; (4) finding motifs enriched in probes related to both differential methylation and target genes; (5) identifying TFs based on transcriptional differences. From this, we obtained a list of 67 motif genes showing methylation differences between INSS stage 4 and 4S NB.

### Signatures based on integrating machine learning algorithms

Based on the methylated differences between INSS stage 4 and 4S NB, we then exploited the INSS stage 4 and 4S related signature (ISRS) using motif genes enriched by ELMER analysis, to diagnose INSS stage 4 NB and predict the prognosis of NB patients. Totally, 67 different methylated genes in motif FOXK1_HUMAN.H11MO.0.A. were included in the construction of machine learning (ML) models. We integrated 10 ML algorithms involving random survival forest (RSF), elastic network (Enet), Lasso, Ridge, stepwise Cox, CoxBoost, partial least squares regression for Cox (plsRcox), supervised principal components (SuperPC), generalized boosted regression modeling (GBM) and survival support vector machine (survival-SVM) to predict prognosis. And we integrated 12 ML algorithms involving random forest (RF), Lasso, Ridge, elastic net (Enet), stepwise Glm, GlmBoost, LDA, partial least squares regression for Glm (plsRglm), GBM, XGB, SVM and Naive Bayes to diagnose stage 4 NB among all NB patients. Altogether 101 prognostic ML combinations and 113 prediction ML combinations were trained in the training cohort, to develop the prognostic and diagnostic models according to the leave-one-out cross-validation (LOOCV) framework. Models with < 5 genes were removed. The GSE49710 cohort was used as the training cohort, then the GSE85047, TARGET-NB, E-MTAB-8248 and E-MTAB-179 cohorts were used as the testing cohorts. Subsequently, the concordance index (C-index) of every ML combination in five cohorts was obtained (Liu et al. 2022). The top five ML combinations yielding the highest average C-index across five cohorts were chosen for further model evaluation via k-fold cross-validation, to mitigate overfitting and ensure the robustness and generalizability of the model. Area under the receiver operating characteristic curve (AUC), area under precision-recall curve (PRAUC), accuracy, sensitivity, specificity, precision, cross-entropy and Brier scores were calculated to identify the best diagnostic ML model via “mlr3” R package (Lang et al. 2019). Precision-recall curve (PRC) was employed to evaluate the performance of classification models in handling imbalanced datasets. Logarithmic loss, recall and decision calibration were utilized to select the best prognostic ML model via “mlr3proba” R package (Sonabend et al. 2021). We incorporated gene expression levels from various feature selection patterns to compute risk scores using a linear combination function for each ML combination, as dictated by the prognostic model. Similarly, we calculated the probability of stage 4 NB using the diagnostic model.

### Verifying the reliability of the diagnostic and prognostic signatures

After selecting the most effective ML pattern pairs, we carried out thorough validation procedures to ensure ISRS with qualified accuracy, consistency, and reproducibility. In the prognostic signature, the median risk score from the training cohort was chosen as the threshold to categorize patients in both the training and validation cohorts into high or low-risk groups. We utilized the Kaplan–Meier (KM) survival analysis and the log-rank test on these groups, using the “survival” and “survminer” R packages. Cox proportional hazards model was utilized to identify the independent prognostic value of prognostic ISRS. In the diagnostic signature, we employed a confusion matrix to validate the precision of the signature via “cvms” R package. Receiver operating characteristic (ROC) curves, calibration curves and decision curve analysis (DCA) were employed to evaluate the precision, discrimination and clinical benefit of the diagnostic and prognostic signatures. Furthermore, we compared the performance of the prognostic signature against traditional clinical variables using time-dependent ROC curves. Additionally, both univariate and multivariate Cox analyses were conducted to affirm the independent predictive power of the prognostic signature.

### Consensus clustering analysis of motif genes

Integrating the model genes from the diagnostic and prognostic signatures, we compiled a set of 24 genes which were used to construct our ML models. Utilizing these genes, we executed unsupervised clustering across three NB cohorts (GSE49710, E-MTAB-8248, and TARGET) using the “ConsensusClusterPlus” R package and the k-means algorithm (Wilkerson and Hayes 2010). This clustering was repeated 1000 times, each time including 80% of the samples. Following this, we visualized the heterogeneity between the two clusters using principal components analysis (PCA) and tSNE plots. To evaluate the efficacy of our clustering analysis, we compared the differences in clinicopathological features and gene expressions between patients of two subtypes using the “ComplexHeatmap” R package. Additionally, survival analysis was conducted to examine the outcomes between the different clusters.

### Functional enrichment analysis

Differentially expressed genes (DEGs) between two molecular subtypes based on consensus clustering analysis, as well as DEGs between two risk groups divided by ISRS, were screened via “limma” R package by False-discovery rate (FDR) < 0.05 and |log2fold change (FC)|> 1. To clarify the biological functions of DEGs between two subtypes and two risk groups, functional enrichment analysis was employed with Gene Ontology (GO) and Kyoto Encyclopedia of Genes and Genomes (KEGG) terms via “clusterprofiler” R package (Yu et al. 2012), and gene set variation analysis (GSVA) was performed with KEGG terms via “GSVA” R package (Hänzelmann et al. 2013). The “h.all.v7.4.symbols.gmt” hallmark gene sets from Molecular Signatures Database (MSigDB) were utilized for GSVA. Gene set enrichment analysis (GSEA) was conducted to identify molecular mechanisms and pathways associated with two subtypes and two risk groups (Subramanian et al. 2005), with the statistical significance threshold set as FDR < 0.25 and Normalized Enrichment Score (NES) > 1.

### Tumor immune microenvironment landscape

Initially, we applied eight distinct algorithms through “IOBR” R package, as well as single sample gene set enrichment analysis (ssGSEA), to measure the levels of immune infiltration in NB patients (Zeng et al. 2021; Newman et al. 2015; Yoshihara et al. 2013; Finotello et al. 2019; Li et al. 2016; Charoentong et al. 2017; Becht et al. 2016; Aran et al. 2017; Racle et al. 2017). Immune cell markers used in ssGSEA were identified from a literature review (Jia et al. 2018). We compared the differences of immune cell abundance between two risk groups divided by ISRS, and between two clusters divided by consensus clustering analysis, based on “Wilcox” test. Besides, we calculate the Spearman correlations between ISRS-predicted risk scores, expressions of model genes and immune cell contents quantified by various immune algorithms. Secondly, we utilized immune function markers identified from the literature review to conduct ssGSEA, assessing and comparing differences in immune function levels between two risk groups, based on “Wilcox” test (Barbie et al. 2009). Thirdly, we conducted ssGSEA to quantify the seven steps of the cancer immunity cycle based on gene markers from the Tracking Tumor Immunophenotype (TIP) website (http://biocc.hrbmu.edu.cn/TIP/) (Xu et al. 2018). Fourthly, we identified 24 inhibitory immune checkpoints from the literature review to compare differences in immune checkpoint gene expressions between two risk groups. Moreover, Thorsson et al. (2018) conducted immunogenomics analyses on over 10,000 cancer samples, identifying six immune subtypes that span a range of cancer subtypes and are believed to characterize immune response patterns, which potentially impact patient prognosis. Five immune subtypes were identified in the GSE49710 cohort, including wound healing (C1), IFN-gamma dominant (C2), inflammatory (C3), lymphocyte depleted (C4) and TGF-β dominant (C6). Subsequently, we focused on the distribution of each immune subtype in two risk groups and two clusters. To analyze the correlation between cancer-associated fibroblasts (CAFs) and immune cells, Estimate the Proportion of Immune and Cancer cells (EPIC), xCell, and Microenvironment Cell Populations-counter (MCP-counter) algorithms were utilized to obtain the CAF scores. And fractions of another 22 immune cells were assessed based on Cell-type Identification By Estimating Relative Subsets Of RNA Transcripts (CIBERSORT) algorithm.

### Somatic mutation and copy number variation analysis

Downloading the somatic mutation data of NB patients from the cBioPortal website (https://www.cbioportal.org/), we evaluated and visualized the mutation types and frequencies of the model genes via “maftools” R package (Mayakonda et al. 2018). In parallel, we calculated the tumor mutation burden (TMB) for each NB patient by determining the total number of somatic mutations per megabase (MB) in the exonic regions of the human genome. Gene mutations were categorized as either synonymous or nonsynonymous mutations. The nonsynonymous mutations involved Frame_Shift_Del, Frame_Shift_Ins, In_Frame_Del, In_Frame_Ins, Missense, Nonsense, Nonstop, Splice_Site, and Translation_Start_Site. We employed GISTIC 2.0 to identify key mutation regions based on copy number variation (CNV) data sourced from the cBioPortal website (Mermel et al. 2011). Additionally, the frequency of somatic CNVs in model genes among NB patients was graphically represented using “bubble plots”, and the chromosomal locations of these mutations were depicted through “circle plots” using the “RCircos” R package (Zhang et al. 2013).

### Evaluation of immunotherapy and chemotherapy sensitivity

To ascertain the effectiveness of ISRS in predicting responses to immunotherapy, we calculated the immune dysfunction and exclusion (TIDE, http://tide.dfci.harvard.edu/) score in two risk groups. Besides, the submap algorithm was employed to assess the response sensitivity of immunotherapy in two risk groups based on a public dataset of immunotherapy (Jiang et al. 2018; Roh et al. 2017). Moreover, four immunotherapy-treated cohorts, IMvigor210, GSE78220, GSE135222, and GSE91061, were obtained to appraise the efficacy of ISRS in predicting responses to immunotherapy. Subsequently, we collected the chemotherapy sensitivity of human cancer cell lines to various drugs from the Cancer Therapeutics Response Portal (CTRP, https://portals.broadinstitute.org/ctrp) website and Profiling Relative Inhibition Simultaneously in Mixtures (PRISM, https://depmap.org/portal/prism/) website. The cell line that is more sensitive to a potential drug would display a lower area under the curve (AUC), which might help screening novel drugs for high-risk NB patients identified by ISRS (Yang et al. 2021).

### Single-cell RNA sequencing analysis

The single-cell RNA sequencing (scRNA-seq) data obtained from the GEO database with accession number GSE137804 was created as Seurat objects via “Seurat” R package (4.1.0) (Satija et al. 2015). We performed quality control to exclude low-quality cells with unique feature counts > 6000 or < 300 or mitochondrial counts > 15%, as well as ribosomes < 3% and erythrocytes < 0.1%, resulting in 172,564 cells finally. We utilized Harmony R package to mitigate technical batch effects while retaining biological variation during multiple batch integration (Korsunsky et al. 2019). The FindVariableFeatures function was performed to identify the top 2000 genes with the highest variation between cells (Butler et al. 2018), on which PCA was performed in the expression matrix. We set resolution at 0.3 to conduct the FindClusters function to identify various clusters. To ascertain the predominant cell type expressing the model genes of ISRS, we performed the RunUMAP function in 4 scRNA-seq datasets (GSE192906, GSE137804, GSE140819 and CellAtlas). We manually annotated the cell type of every cluster based on the expression of canonical markers from the literature review (Dong et al. 2020). Six scoring algorithms (AUCell in “AUCell” R package, Ucell in “Ucell” R package, ssGSEA in “GSVA” R package, singscore in “singscore” R package, AddModuleScore and PercentageFeatureSet in “Seurat” R package) were utilized to perform signature enrichment scoring in four NB scRNA-seq datasets (Hänzelmann et al. 2013; Satija et al. 2015; Andreatta and Carmona 2021; Foroutan et al. 2018; Aibar et al. 2017), which were visualized with “irGSEA” R package (https://chuiqin.github.io/irGSEA/index.html). Pseudotime trajectory analysis was conducted through “Monocle” R package and “Monocle3” R package to explore map conversion trajectories without prior knowledge of differentiation time or direction (Qiu et al. 2017; Cao et al. 2019). “InferCNV” R package was utilized to assess CNVs in neuroendocrine (NE) cells, Schwann cells, endothelial cells, and fibroblasts. T cells, B cells and myeloid cells were considered as references to assess CNVs in cancerous cells (Tirosh et al. 2016). “CellChat” R package was used to explore the intercellular communication patterns between each cell type (Jin et al. 2021). Meanwhile, “pySCENIC” (version 0.11.2) in Python (version 3.7) was performed to investigate TF enrichment and regulon activity, which aided in the construction of TF regulatory networks and the identification of stable cell states (Aibar et al. 2017).

### Pan-cancer analysis

We performed pan-cancer analysis via “TCGAplot” R package to explore hub genes’ similarities and differences in genomic and cellular changes across a variety of tumor types (Liao and Wang 2023), in terms of gene expression, TMB, microsatellite instability (MSI), immune microenvironment and prognostic value. Gene expressions in tumor and normal samples were compared by “Wilcox” test, while the correlation between the gene expression and TMB, MSI, immune cell and immune score was calculated by the Spearman method. Immune cell ratio was retrieved from The Immune Landscape of Cancer (https://api.gdc.cancer.gov/data/b3df502e-3594-46ef-9f94-d041a20a0b9a), and immune scores were analyzed by the ESTIMATE algorithm.

### Immunohistochemistry staining

To validate the different expressions of model genes between stage 4 and 4S NB, we conducted immunohistochemistry (IHC) staining of two model genes (CAMTA2 and FOXD1) in 25 stage 4 NB tissues and 10 stage 4S NB tissues. The experiment received approval from the ethics committee of the Children’s Hospital of Chongqing Medical University. NB tissues were paraffin-embedded and cut into 4 mm slices. Following dewaxing, hydration, and antigen retrieval, the samples were treated with primary antibodies: Anti-CAMTA2 (Affinity Biosciences Cat# DF9314, RRID: AB_2842510) and Anti-FOXD1 (bs-12193R, Bioss, Beijing, China), and incubated overnight at 4 °C. Subsequent steps included incubation with Goat anti-Rabbit IgG secondary antibody (ZENBIO, China), DAB staining (ZENBIO, China), and blocking, with the staining effects observed under a microscope. Each sample was scored based on staining intensity (0: none, 1: mild, 2: moderate, 3: strong) and the percentage of positive cells (0: 0%, 1: 1–25%, 2: 26–50%, 3: 51–75%, 4: 76–100%). The final IHC score was calculated as the sum of both intensity and percentage scores.

## Results

### Enhancer-associated regulatory network with ELMER analysis

**Fig. 2:**
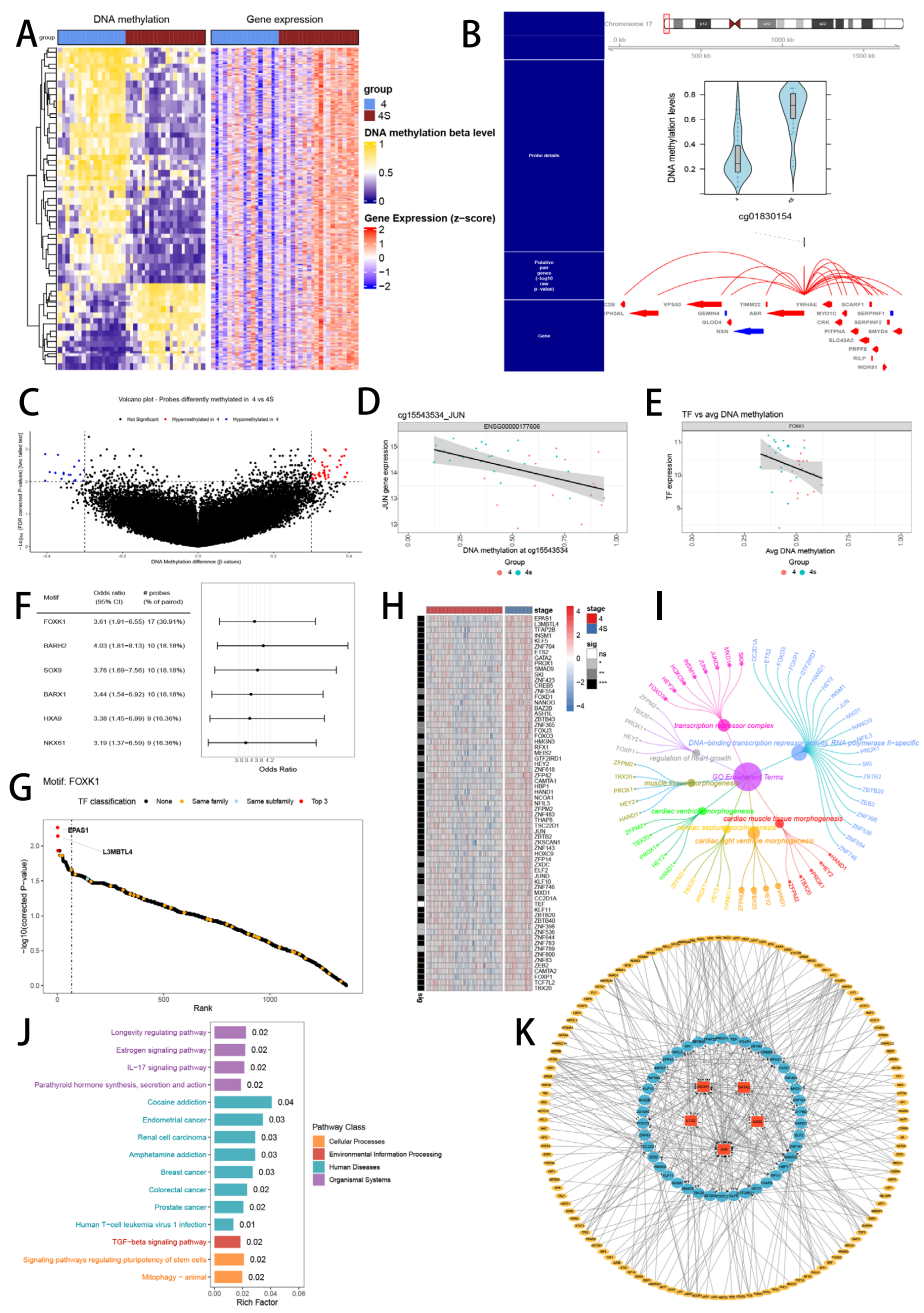
Enhancer-associated regulatory network was constructed with ELMER. **A** The heatmap visualized the differences in methylation and transcriptomic levels between INSS 4 and 4S stages in the GSE73518 cohort. Color gradient: gene expression levels (blue to red, blue for low, red for high) and corresponding methylation levels (steelblue to yellow, steelblue for low, yellow for high). **B** Example of top ten genes closest to the upstream and downstream of the differentially methylated distal probes. **C** Volcano plot of probes that were hypermethylated and hypomethylated in INSS 4 stage NB patients. **D** Example of correlation plot between the TF expression level and DNA methylation level of one of its probes. **E** Example of correlation plot between the TF expression level and its corresponding average DNA methylation level. **F** Odds ratios of the significantly enriched motifs identified by the get.enriched.motif function. **G** TF sorting plot of motif FOXK1_HUMAN.H11MO.0.A. **H** Profiles of gene expressions of motif FOXK1_HUMAN.H11MO.0.A. in stage 4S and stage 4. Color gradient: gene expression levels (blue to red, blue for low, red for high). **I**–**J** Function enrichment performed by GO and KEGG terms focused on motif FOXK1_HUMAN.H11MO.0.A. **K** TF regulatory network of motif FOXK1_HUMAN.H11MO.0.A visualized with Cytoscape software

Methylation and transcriptomic data of the same patient, particularly in 17 INSS stage 4 NB patients (age < 1.5 years) and 20 INSS stage 4S NB patients from the GSE73518 cohort, were incorporated in ELMER analysis. We plotted a heatmap to visualize the variations of methylation and transcriptomic levels between stage 4 and 4S NB (Fig. 2A). Distal probes were used to detect the region of enhancers. According to the hg38 reference genome, we selected 135,427 distal probes by get.feature.probe function (Supplementary Table S3). We utilized the GetNearGenes function to pinpoint the top ten genes nearest to the upstream and downstream of the distal probes respectively, thus creating probe-gene pairs (Fig. 2B). Subsequently, an unsupervised approach was utilized to detect distal probes by get.diff.meth function. Each probe’s methylation levels were ranked in both stage 4 and 4S groups, and the samples in the lower or higher quintiles (20%) of methylation were analyzed to determine whether the probes were hypo- or hypermethylated in stage 4, leading to the identification of 57 distal probes (Supplementary Table S4) with an FDR < 0.01 and a |Δ*β*| < 0.3 (Fig. 2C). We then examined the inverse correlation between each probe's methylation level and its associated gene's expression. Samples were classified into methylated (M) and unmethylated (U) groups based on the top and bottom 20% methylation levels of the probes, and gene expression levels between these groups were compared using the Mann–Whitney *U* test. Unsupervised mode was utilized to screen 956 probe-gene pairs with statistically significant negative correlations with default parameters through get.pair function (Supplementary Table S5). Then, the 250 bp base sequence upstream and downstream of these probes were extracted and aligned to 37 significantly enriched motifs (Supplementary Table S6) by get.enriched.motif function. Distal probes associated with the same motif were then classified into the top 20% M and bottom 20% U groups based on methylation levels. Motif FOXK1_HUMAN.H11MO.0.A. with differential expressions between two stages and negative correlations with its methylation levels was obtained through get.TFs function via the unsupervised mode (FDR < 0.05) (Fig. 2D–F, Supplementary Table S7) (Lambert et al. 2018). To improve the robustness of the identified regulatory networks, we also employed a supervised mode for probe selection and motif identification, which revealed that motif FOXK1_HUMAN.H11MO.0.A. enriched differentially between two stages, with significance (FDR < 0.05) (Supplementary Table S8). Ranking the TFs in motif FOXK1_HUMAN.H11MO.0.A. discovered that EPAS1 and L3MBTL4 may play a vital role in methylated regulation (Fig. 2G). Besides, most of the genes in motif FOXK1_HUMAN.H11MO.0.A. expressed significantly higher in stage 4S than in stage 4 (Fig. 2H). GO and KEGG enrichment analysis showed that motif genes may affect embryonic organ development, muscle tissue development, morphogenesis and signaling pathways regulating pluripotency of stem cells (Fig. 2I, J). The enhancer-associated regulatory network is visualized via Cytoscape software (Fig. 2K).

### Establishment and validation of ISRS with diagnostic and prognostic value

**Fig. 3:**
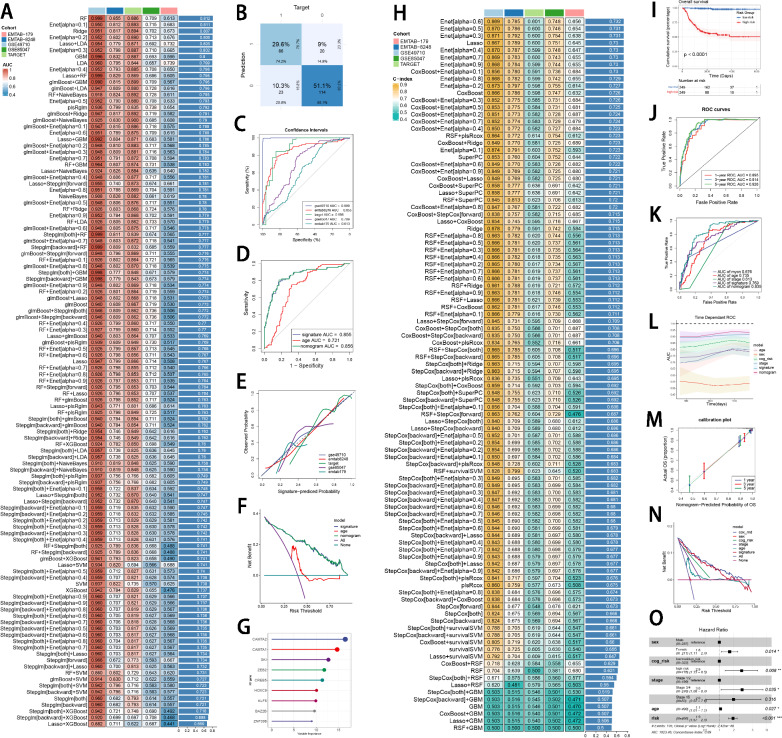
Construction and validation of a diagnostic and a prognostic signature based on 67 genes selected by ELMER analysis. **A** A total of 113 kinds of diagnostic models via a leave-one-out cross-validation framework and further calculated the C-index of each model. **B** Confusion matrix of the diagnostic ISRS in the validation cohort E-MTAB 8248. **C** ROC curves of the diagnostic ISRS in five cohorts (GSE49710, E-MTAB 8248, TARGET, GSE85047 and E-MTAB 179). **D** ROC curves of the diagnostic ISRS, the logistic regression model (nomogram) and several clinical variables in the validation cohort E-MTAB 8248. **E** Calibration curves of the diagnostic ISRS in five cohorts (GSE49710, E-MTAB 8248, TARGET, GSE85047 and E-MTAB 179). **F** DCA curves of the diagnostic ISRS, the logistic regression model (nomogram) and several clinical variables in the validation cohort E-MTAB 8248. **G** The feature importance visualization of 9 variables selected by RF, which formed the final diagnostic model. **H** A total of 101 kinds of prognostic models via a leave-one-out cross-validation framework and further calculated the C-index of each model. **I** Kaplan–Meier survival curves of OS for high-risk and low-risk groups of NB patients in the GSE49710 cohort. **J** ROC curves of 1-, 3- and 5-year OS of the prognostic ISRS signature in the GSE49710 cohort. **K** AUC values of 3-year OS of the prognostic ISRS signature, the cox regression model (nomogram) and several clinical variables in the GSE49710 cohort. **L** Time-dependent ROC curves of the prognostic ISRS signature, the cox regression model (nomogram) and several clinical variables in the GSE49710 cohort. **M** 1-, 3- and 5-year calibration curves of the prognostic signature in the GSE49710 cohort. **N** DCA curves of the prognostic ISRS signature, the cox regression model and several clinical variables in the GSE49710 cohort. **O** Forest plot visualized the outcome of multivariate Cox regression analysis involving the prognostic ISRS and several clinical variables. Risk: risk scores calculated by prognostic ISRS

As mentioned above, five NB cohorts with transcriptome data were utilized in model development and verification, which showed a superior effectiveness of batch effect removal via “sva” R package (Supplementary Figure S1A–B). Incorporating 67 genes in motif FOXK1_HUMAN.H11MO.0.A. based on ELMER analysis, 101 prognostic algorithm combinations and 113 prediction algorithm combinations were then constructed via the LOOCV framework. The C-index of each ML combination was calculated in all validation datasets (Supplementary Table S9). The best diagnostic model was established based on RF algorithm which was utilized in both feature selection and model construction, with the highest average C-index (0.812) across five datasets (Fig. 3A). AUC, PRAUC, accuracy, sensitivity, specificity, precision, cross-entropy and Brier scores were utilized to reveal that RF was the most powerful model in diagnosing INSS 4 NB (Supplementary Figure S1C–D). Finally, a 9-gene diagnostic INSS stage-related signature (ISRS) was accordingly established to diagnose stage 4 NB, with CAMTA2 being the most important variable (Supplementary Figure S1E–F). Confusion matrix in E-MTAB 8248 cohort and other three validation cohorts showed a well accuracy of ISRS (Fig. 3B, Supplementary Figure S2A). ROC curves of five cohorts showed a well discrimination of ISRS (Fig. 3C). We also compared the AUC of ISRS, other clinical variables and the logistic regression nomogram model including several clinical variables and ISRS, which demonstrated that ISRS and the nomogram model performed better (Fig. 3D, Supplementary Figure S2B–C). Calibration curves showed a great alignment between ISRS predicted probability and the observed probability of stage 4 NB (Fig. 3E). DCA curves indicated that utilization of ISRS and the logistic regression nomogram model is more clinically beneficial, compared with other clinical variables (Fig. 3F, Supplementary Figure S2D). The feature importance visualization of 9 variables selected by RF demonstrated that CAMTA2 impacted most in the RF model (Fig. 3G). The best prognostic model was established based on the Enet (alpha = 0.6) algorithm which was utilized in both feature selection and model construction, with the highest average C-index (0.732) across five datasets (Fig. 3H). Logarithmic loss, recall and decision calibration were calculated to prove the well calibration and precision of the Enet (alpha = 0.6) model (Supplementary Figure S1G). Ultimately, a 20-gene prognostic ISRS was accordingly established to forecast the prognosis of NB patients (Supplementary Figure S1H–I). The feature importance visualization of 20 variables selected by Enet demonstrated that CAMTA1 impacted most in the Enet model (Supplementary Figure S1J). In GSE49710, E-MTAB 8248, TARGET and E-MTAB 179 cohorts, the low-risk group owned a relatively longer overall survival (OS) and event-free survival (EFS) than the high-risk group (Fig. 3I, Supplementary Figure S3A). In GSE85047 cohort, the low-risk group owned a relatively longer overall survival (OS) and progression-free survival (PFS) than the high-risk group (Supplementary Figure S3A). ROC curves of 1-, 3- and 5-year OS showed well specificity of ISRS (Fig. 3J, Supplementary Figure S3B). AUC values of 3-year OS proved that ISRS and cox regression model involving ISRS and several clinical variables were more specific and discriminative to forecast the prognosis of NB patients, compared to other clinical variables (Fig. 3K, Supplementary Figure S3C–D). Time dependent ROC curves indicated that ISRS and cox regression model outperformed conventional clinical variables in capability of discrimination (Fig. 3L, Supplementary Figure S3E). Calibration curves (Fig. 3M, Supplementary Figure S3F) and DCA curves (Fig. 3N, Supplementary Figure S3G) showed that ISRS is well-behaved in accuracy and clinical benefit. Multivariate Cox regression analysis in the Cox proportional hazards model indicated that ISRS, age, sex, INSS stage and COG risk stratification were independent prognostic factors in NB patients (*P* < 0.05) (Fig. 3O, Supplementary Figure S3H). All these metrics collectively indicated that ISRS demonstrated stability and robustness in model performances across five NB queues. The flowchart of machine learning algorithm integration was provided in Supplementary Figure S1K.

### Functional enrichment analysis and landscape of model genes

**Fig. 4:**
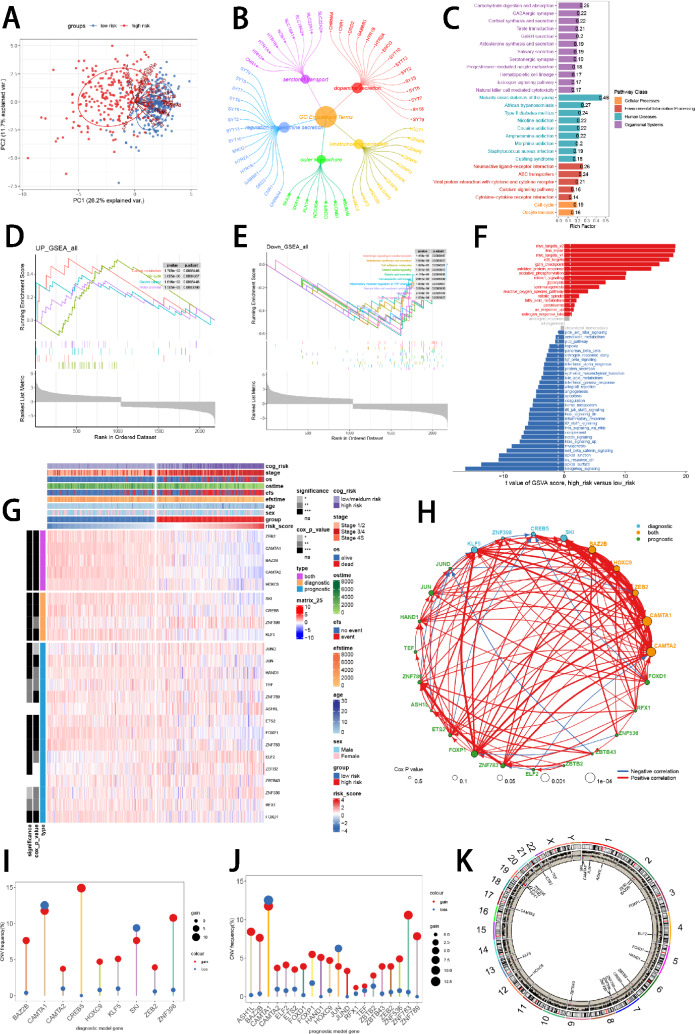
Functional enrichment analysis and landscape of model genes. **A** PCA analysis plot of high-risk group and low-risk group. **B**, **C** GO and KEGG enrichment analyses of DEGs among two risk groups. **D**–**F** GSEA and GSVA analyses of DEGs among two risk groups. **G** Differences in the expression of model genes and differences in the clinical variables of NB patients among the two risk groups. **H** Molecular interaction network plot visualized the correlation among expressions of model genes and their prognostic prediction value. Significantly positive and negative correlations are shown as red and blue lines, respectively. The color and size of the nodes indicate the type of model genes and *P* values from Cox regression. **I**, **J** The CNV mutation frequency of the diagnostic model genes and the prognostic model genes. **D** Chromosome position and alteration of all model genes

PCA analysis displayed significant variations between two risk groups divided by prognostic ISRS (Fig. 4A). Based on 7325 DEGs identified between two risk groups via “limma” R package, we performed functional enrichment analysis according to GO and KEGG terms, which discovered that DEGs were significantly enriched in dopamine secretion, kinetochore organization, serotonin transport and outer kinetochore in GO terms, and were significantly enriched in Neuroactive ligand-receptor interaction, Cell cycle, GABAergic synapse and Calcium signaling pathway in KEGG terms (Fig. 4B, C). GSEA analysis demonstrated that carbon metabolism and cell cycle were enriched in a high-risk group, and adrenergic signaling in cardiomyocytes, aldosterone synthesis and secretion and cell adhesion molecules were suppressed in a high-risk group (Fig. 4D, E). To understand the biological behavioral variations between two risk groups, we conducted GSVA enrichment analysis based on “h.all.v7.4.symbols.gmt” hallmark gene sets in MSigDB database, which indicated that high-risk group was enriched in myc_targets_v2 and DNA_repair, and suppressed in hedgehog_signaling and apical_surface (Fig. 4F). We then visualized the differential expression of ISRS model genes and the variations of clinical variables between the two risk groups (Fig. 4G). ZEB2, CAMTA1, BAZ2B, CAMTA2 and HOXC9, which constituted both the diagnostic and prognostic ISRS, were significantly higher expressed in a low-risk group than in a high-risk group, and significantly higher expressed in stage 4S than in stage 4, pretending to be protectively prognostic genes (Supplementary Figure S4A-B). Detailed information on types of model genes was provided in Supplementary Table S7. Spearman correlation analysis revealed close relationships (correlation *P* value < 0.0001) among the 24 ISRS model genes (Fig. 4H). Based on CNV data sourced from the cbioportal website, “bubble plots” visualized that CREB5 attained the most somatic CNV frequencies among diagnostic model genes, and CAMTA1 attained the most somatic CNV frequencies among prognostic model genes (Fig. 4I, J). Besides, “circle plots” visualized the chromosome locations where the mutations of ISRS model genes occurred (Fig. 4K).

### Tumor immune microenvironment analysis

**Fig. 5:**
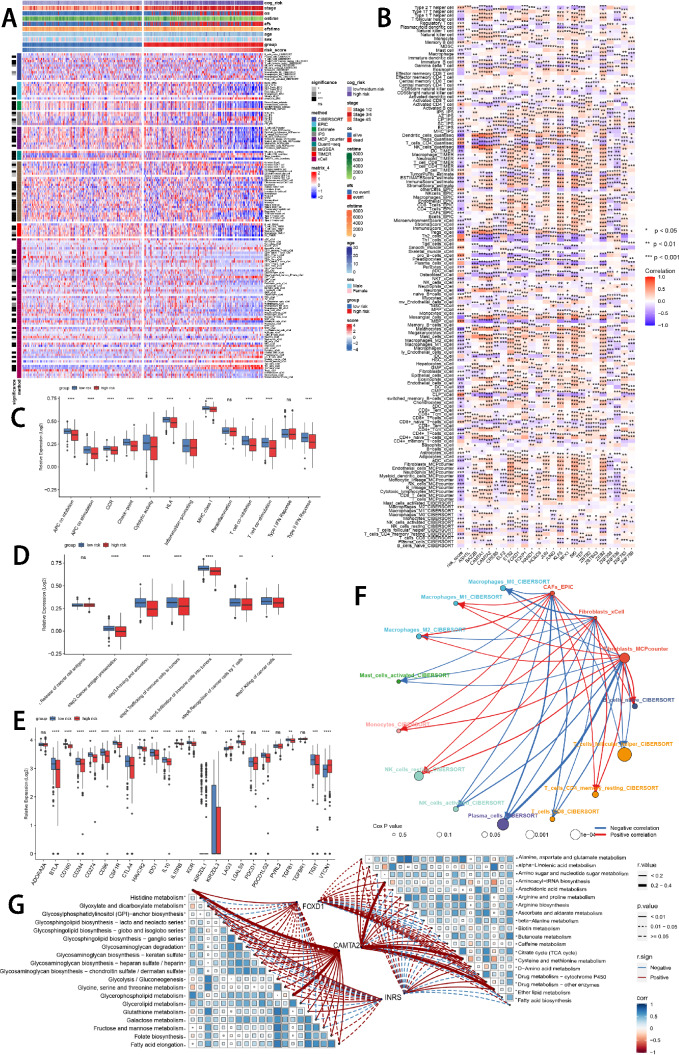
Analysis of the TME in different risk groups. **A** Differences in immune infiltration status between the two risk groups were evaluated by eight immune algorithms. **B** Heatmap visualized the correlation between different immune cells and risk scores and the relationship between different immune cells and expressions of model genes. **C** The differences in immune function scores were calculated by ssGSEA analysis between the two risk groups. **D** The differences in cancer immunity cycle scores based on ssGSEA analysis between two risk groups. **E** The differences in expressions of immune checkpoint-related genes between two risk groups. **F** Network showed the correlation among CAFs and CIBERSORT-derived immune cells. Significantly positive and negative correlations are shown as red and blue lines, respectively. The color and size of the nodes indicate the type of immune cells and *P* values from Cox regression. **G** The correlations between the signature risk scores, expressions of FOXD1 and CAMTA2, and metabolic-related pathways based on GSVA analysis of KEGG terms were displayed in the butterfly plot

To appraise the discriminative capability of the prognostic ISRS in immune infiltration, we simultaneously calculated the abundance of immune cell infiltration based on eight distinct immune algorithms. “ComplexHeatmap” R package visualized that various immune cells were significantly fewer infiltrated in a high-risk group than in the low-risk group (Fig. 5A). Besides, the spearman correlation heatmap displayed the correlation between immune cell infiltrations and the risk scores calculated by the prognostic ISRS, as well as the relationship between immune cell infiltrations and the expression of ISRS model genes (Fig. 5B). Besides, ssGSEA analysis of immune function scores demonstrated that the low-risk group owned significantly better immune infiltration abundance (Fig. 5C). Moreover, the activation of six key steps in the cancer immunity cycle appeared to be significantly higher in the low-risk group (Fig. 5D). For immune checkpoints, the low-risk group displayed elevated expression levels of immune checkpoint genes, indicating a susceptibility to immunotherapy (Fig. 5E). Previous literatures have stressed the vital role of cancer-associated fibroblasts (CAFs) in immune modulation of the tumor microenvironment (Boyle et al. 2020; Sahai et al. 2020; Gagliano et al. 2020). We then utilized “circle plot” to visualized the correlation of CAFs among various types of immune cells calculated by the CIBERSORT algorithm in NB patients (Fig. 5F). Additionally, it is well-known that heterogeneous metabolic preferences and dependencies exist across tumor types (Hakimi et al. 2016; Hensley et al. 2016; Kim and DeBerardinis 2019), hence we extracted multiple metabolic pathways from the KEGG database to validate the relationship between risk scores and tumor metabolism in NB patients (Fig. 5G). Two hub model genes (FOXD1 and CAMTA2) also showed significant correlations with cell metabolic pathways.

### Comparison of the tumor mutation burdens

**Fig. 6:**
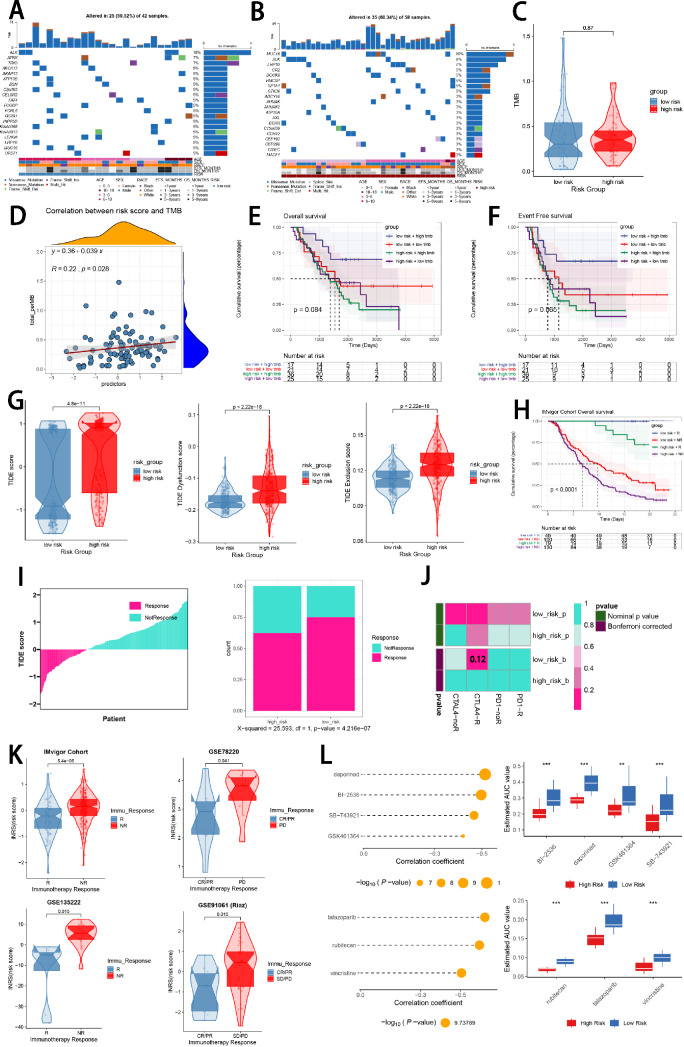
Landscape of somatic mutation, CNVs, immunotherapy and chemotherapy between high-risk and low-risk groups. **A**, **B** Visual summary displayed common genetic alterations in the high-risk and low-risk groups. **C** Tumor mutation burdens between high-risk and low-risk groups. **D** Spearman correlation between risk scores calculated by ISRS and TMB scores in NB patients. **E**, **F** Comprehensive survival analysis on OS and EFS based on two risk groups and two TMB groups. **G** Violin diagram illustrated the variance in TIDE scores between high-risk and low-risk groups in the GSE49710 cohort. **H** Kaplan–Meier survival analysis delineated the OS rates for patients categorized into high-risk and low-risk groups in the IMvigor cohort. **I** The TIDE algorithm predicted response to immunotherapy between high-risk and low-risk groups in the E-MTAB 8248 cohort. **J** Comprehensive submap analysis predicted response to immunotherapy between high-risk and low-risk groups in the E-MTAB 8248 cohort. **K** Box diagram depicted the disparity in ISRS among patients exhibiting immunotherapy responses in the IMvigor210, GSE78220, GSE135222, and GSE91061 cohorts. **L** Correlation study and differential drug response analysis of CTRP-derived pharmaceuticals and PRISM-derived pharmaceuticals to explore potential drugs for high-risk NB patients

We visualized and compared the distribution variations of somatic mutations between the low-risk group (Fig. 6A) and the high-risk group (Fig. 6B) based on mutation data sourced from the cbioportal website. Comparisons of tumor mutation burden (TMB) between the two risk groups revealed no significant difference (Figs. 6C). However, TMB was found to be significantly related to the risk scores obtained by the prognostic ISRS based on spearman correlation analysis (Fig. 6D). Moreover, we classified NB patients with mutation data into the high TMB and the low TMB group according to their median TMB score. After integrating two risk groups and two TMB groups, we found that patients with low TMB in the high-risk group owned the worst OS and EFS, and patients with high TMB in the low-risk group owned the best OS and EFS, without significance (Fig. 6E, F).

### Response of immunotherapy and chemotherapy

Based on TIDE scores and submap algorithm, the possibility of immunotherapy responses was appraised in two risk groups. In the GSE49710 cohort, higher TIDE scores, higher TIDE dysfunction and exclusion scores were discovered in the high-risk group, indicating a bigger probability of immune escape during the immunotherapy process (Fig. 6G). In the IMvigor210 immunotherapy cohort, survival analysis demonstrated that low-risk patients with a response to immunotherapy owned significantly longest OS, whereas high-risk patients with no response to immunotherapy had significantly worst OS (Fig. 6H). Meanwhile, in the E-MTAB8248 cohort, a significantly higher proportion of patients responding to immunotherapy was observed in the low-risk group (Fig. 6I). Besides, the submap analysis results demonstrated that low-risk patients were more sensitive to CTLA4 inhibitors, rather than PD1 inhibitors (Fig. 6J). Despite we assessed individual immunotherapy reaction based on two methods in two NB cohorts, it's crucial to straightly compare treatment effectiveness in immunotherapy-treated cohorts between two risk groups, which called a need to involve four immunotherapy-treated cohorts in subsequent analysis. Ultimately, we found that patients who responded more sensitively to immunotherapy owned significantly lower risk scores across four immunotherapy-treated cohorts (Figs. 6K). Accordingly, to explore potential drugs that might be more effective in high-risk NB patients, we predicted drug response based on drug sensitivity data from CTRP and PRISM. Through the cross-correlation in the two pharmacogenomics databases, we successfully predicted six potential drugs or compounds (BI-2536, daporinad, GSK461364, SB-743921, rubitecan and talazoparib) with therapeutic effects in high-risk NB patients (Fig. 6L).

### Identification of ISRS model genes-related subtypes

**Fig. 7:**
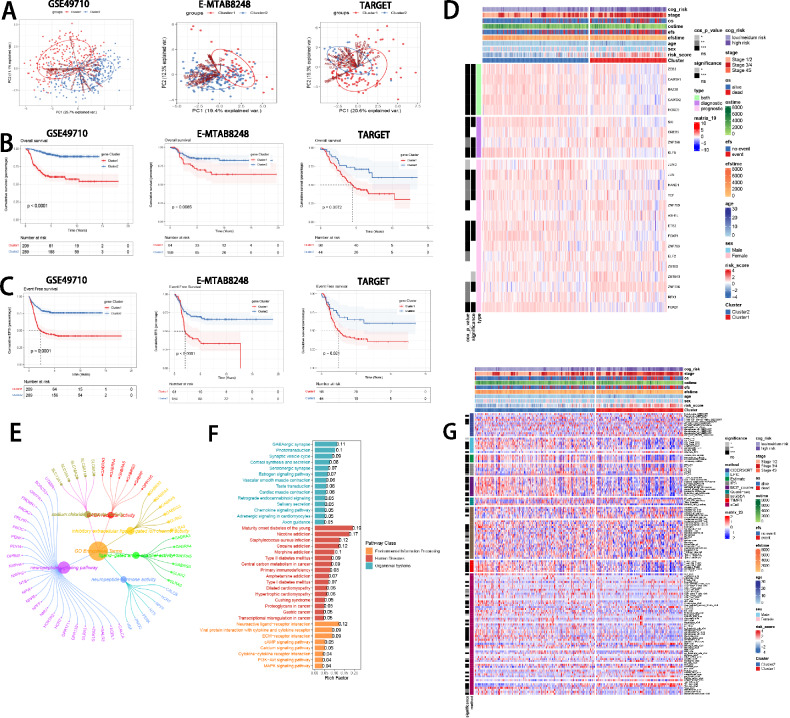
Construction of ISRS model genes-related clusters. **A** PCA analysis of two clusters. **B**, **C** Kaplan–Meier survival analysis of OS and EFS between two clusters. **D** ComplexHeatmap of the distribution of ISRS model genes and clinical variables in the two clusters. **E**, **F** GO and KEGG enrichment analysis indicated significant enrichment of pathways in cluster 1. **G** Differences in the proportion of various kinds of immune cells calculated by eight immune algorithms in the two clusters

To further understand the expression pattern of ISRS model genes, 871 patients from GSE49710, E-MTAB 8248 and TARGET cohorts were included for consensus clustering analysis. Respectively, we utilized ISRS model genes to employ unsupervised clustering analysis on each cohort, which demonstrated that *k* = 2 exhibited the best discrimination (Supplementary Figure S5A–B). Besides, PCA and tSNE analysis showed significant variations between the two clusters, according to the expressions of ISRS model genes (Fig. 7A, Supplementary Figure S5C). Meanwhile, we performed survival analysis to demonstrate that cluster 1 owned significantly worse OS and EFS than cluster 2 (Fig. 7B, C). Moreover, the expressions of ISRS model genes and the clinicopathological characteristics in different clusters displayed significant differences (Fig. 7D). Based on 1868 DEGs identified between two clusters via “limma” R package, we performed functional enrichment analysis, which discovered that DEGs were significantly enriched in neuropeptide signaling pathway, neuropeptide hormone activity, ligand-gated ion channel activity, sodium:chloride symporter activity and GABA receptor activity in GO terms, and were significantly enriched in Neuroactive ligand-receptor interaction, ECM-receptor interaction, GABAergic synapse, Synaptic vesicle cycle and Neomycin, kanamycin and gentamicin biosynthesis in KEGG terms (Fig. 7E, F). GSEA analysis demonstrated that cytokine-cytokine receptor interaction and staphylococcus aureus infection were enriched in cluster 1, and GABAergic synapse and neuroactive ligand-receptor interaction were suppressed in cluster 1 (Supplementary Figure S5D). Moreover, GSVA enrichment analysis, based on “h.all.v7.4.symbols.gmt” hallmark gene sets in the MSigDB database, indicated that cluster 1 was enriched in myc_targets_v2, e2f_targets and g2m_checkpoint, and suppressed in hedgehog_signaling and apical_junction (Supplementary Figure S5E). To further understand variations in tumor immune microenvironment between two clusters, eight immune algorithms were utilized to assess the immune abundance differences between two clusters, and visualized the Cox *P* value of each immune cell type (Fig. 7G). In terms of mutation landscape, we compared the distribution variations of somatic mutations between cluster 1 (Supplementary Figure S5F) and cluster 2 (Supplementary Figure S5G) based on mutation data sourced from the cbioportal website. We observed that TMB scores are not significantly different between the two clusters (Supplementary Figure S5H). Meanwhile, we classified NB patients with mutation data into the high TMB and the low TMB group according to the median TMB score. After integrating two clusters and two TMB groups, we found that patients with low TMB from cluster 1 had worse OS and EFS than patients with high TMB from cluster 2, without significance (Supplementary Figure S5I–J).

### Model comparisons and landscapes of risk groups and clusters

**Fig. 8:**
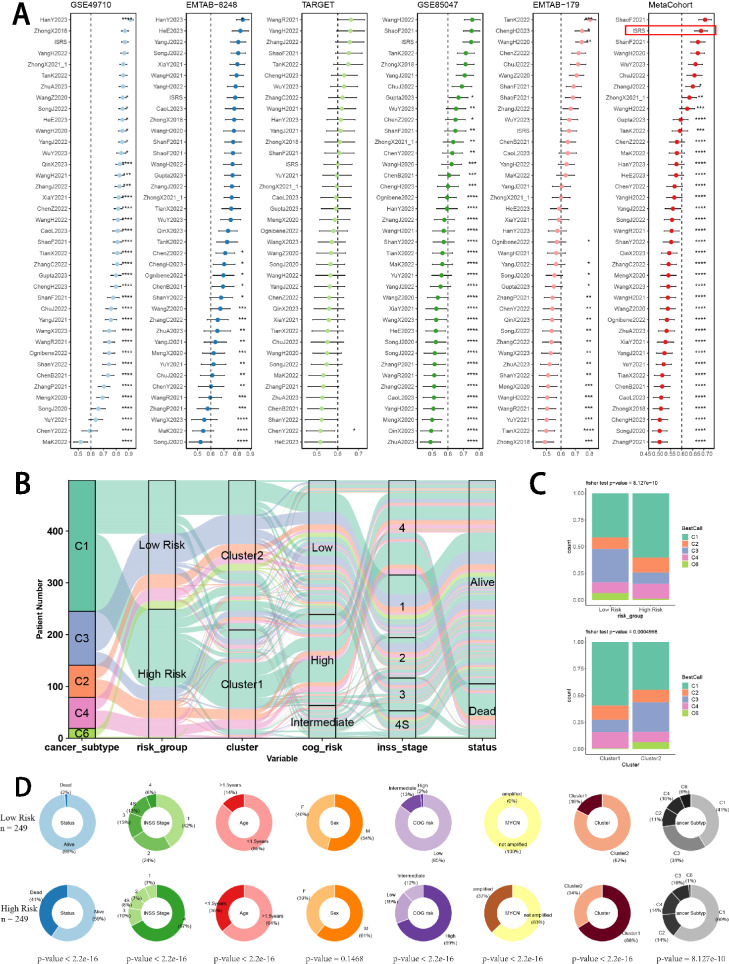
Model comparisons and landscape of two risk groups and two clusters. **A** C-index comparison analysis between the ISRS and 39 published signatures in GSE49710, E-MTAB 8248, TARGET, GSE85047, E-MTAB 179 and meta-cohort. *P < 0.05; **P < 0.01; ***P < 0.001; ****P < 0.0001. **B** Sankey diagram of distributions in two clusters and two risk groups with different clinical variables and survival outcomes. **C** Differences in the proportion of five immune subtypes between two clusters and two risk groups. **D** Circular pie chart visualized the proportion difference of clinical indices and immune subtypes between two risk groups

For the sake of verifying the prognostic effectiveness of ISRS, we collected coefficients of model genes in 39 previously published NB prognostic models. Subsequently, we performed a comparative analysis of the C-index of each prognostic model, which was developed based on a variety of biological characteristics, involving necroptosis, ferroptosis, cuproptosis, disulfidptosis, ganglioside, and m6A methylation. Ultimately, we discovered that ISRS demonstrated superior predictive performances relative to the vast majority of models across five NB datasets (Fig. 8A), which qualified ISRS as an invaluable NB prognostic model. Moreover, the overall relationship among different risk groups, different clusters and patients’ clinical information was visualized by “sankey plot” (Fig. 8B). In terms of immune subtypes of NB patients, we observed significant variations in the proportion of immune subtypes between two risk groups and two clusters in the GSE49710 cohort, with much more wound healing (C1) subtype in high-risk group and in cluster 1 (Fig. 8C). Comparing clinical variables between two risk-groups, we suggested that lower risk scores of ISRS were related to better prognoses in NB patients (Fig. 8D).

### Single-cell sequencing analysis and pseudotime analysis

**Fig. 9:**
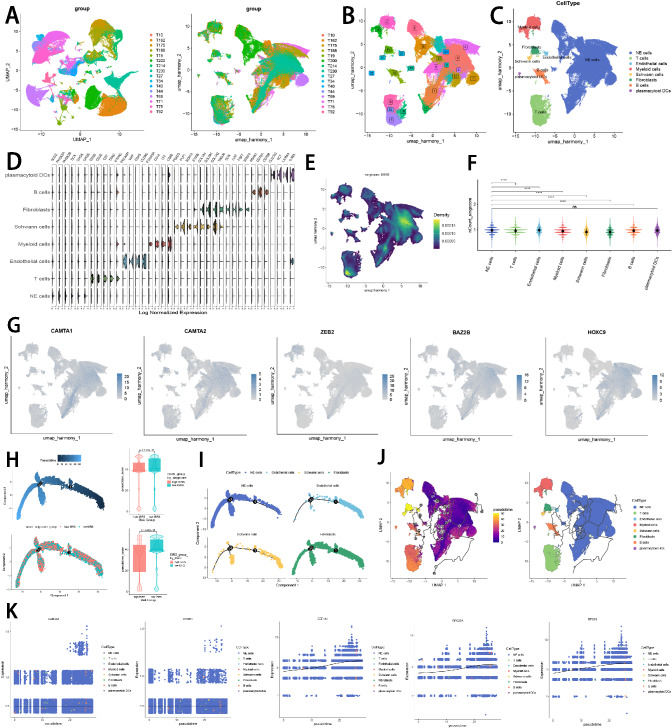
Exploration of ISRS and model genes in GSE137804 scRNA-seq data. **A** UMAP plot before and after Harmony integration, each color stands for a NB tumor sample. **B**, **C** UMAP plot of the distribution of 24 clusters and 7 cell types after manual annotation. **D** Violin plot of maker genes for annotation of each cell type. **E**, **F** Single-cell scoring results of ISRS model genes based on the singscore algorithm in each cell type. **G** FeaturePlot of ISRS model genes (CAMTA1, CAMTA2, ZEB2, BAZ2B and HOXC9) in UMAP plot. **H**–**I** Pseudotime trajectory analysis in NB cells via Monocle 2 algorithm (Cells are colored according to pseudotime, ISRS groups and cell types). **J**–**K** Pseudotime trajectory analysis in NB cells and pseudotime DEGs via Monocle 3 algorithm

To explore the biological function of ISRS in a single cell level, we utilized 4 NB single-cell datasets (GSE137804, GSE192906, GSE140819 and CellAtlas) to validate our results. In GSE137804, we utilized harmony integration to remove batch effects, which showed a well correction before and after integration (Fig. 9A). Subsequently, we partitioned all cells in GSE137804 into 25 clusters based on the k-nearest neighbor (KNN) clustering method (Fig. 9B). According to cell markers collected from the literature review, we successfully identified 8 distinct cellular subtypes, involving neuroendocrine cells (NE cells), T cells, B cells, endothelial cells, myeloid cells, Schwann cells, fibroblasts, and plasmacytoid DC cells (Fig. 9C). Violin plot visualized the expressions of canonical markers in 8 clusters (Fig. 9D). Subsequently, the ISRS scoring for each cell was calculated based on six signature enrichment scoring algorithms, which demonstrated that cells with higher ISRS scoring predominately located in NE cells (Fig. 9E, F, Supplementary Figure S6A–B). Meanwhile, we explored the single-cell transcriptome localization of ISRS model genes, which were found to expressed mostly in NE cells (Fig. 9G). Accordingly, the expression patterns of ISRS model genes were validated in three NB single-cell external datasets (GSE192906, GSE140819 and CellAtlas) (Supplementary Figure S6C). Meanwhile, six scoring algorithms were utilized in the three NB single-cell external datasets (GSE192906, GSE140819 and CellAtlas) to demonstrate that ISRS model genes were enriched in NE cells (Supplementary Figure S6D–I). Additionally, the pseudotime trajectory analysis revealed the temporal sequence of malignant cellular differentiation and demonstrated that NE cells with higher ISRS scores appeared at an earlier pseudotime than NE cells with lower ISRS scores, indicating that immature NE cells were more predominant in high-risk NB patients (Fig. 9H). The differentiation trajectory of NE cells, endothelial cells, fibroblasts and Schwann cells were plotted via Monocle 2 algorithm, which suggested that endothelial cells owned the potential to differentiate into other types of cells (Fig. 9I). Hence, we set endothelial cells as the beginning of differentiation to investigate pseudotime trajectory of all cells in Monocle 3 analysis, which validated that immature NE cells could own higher ISRS scores and serve as malignant cells (Fig. 9J). Besides, pseudotime analysis of interested genes identified that pseudotime DEGs were increased along the pseudotime curve, while the ISRS model genes stayed still (Fig. 9K).

### Validation of ISRS by inferCNV, cell communication and SCENIC analysis

**Fig. 10:**
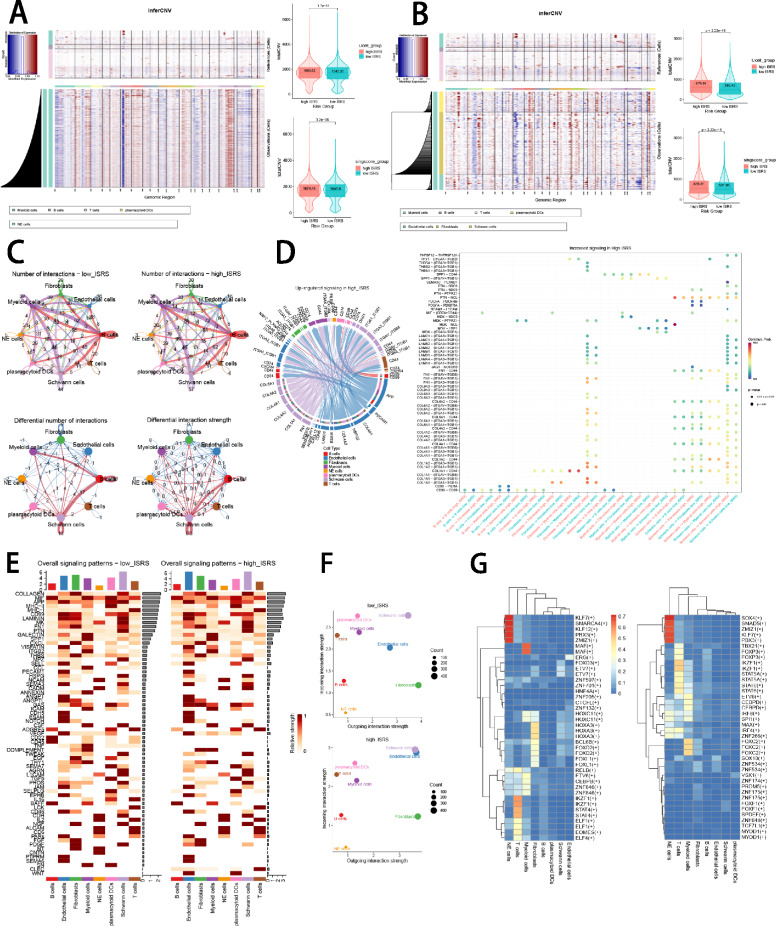
The landscape of CNV, cell–cell communication, transcriptional regulons in GSE137804 scRNA-seq cohort. **A**, **B** Differences of CNVs of NE cells, fibroblasts, Schwann cells and endothelial cells in high ISRS cells and low ISRS cells. **C** Circle diagrams showed the interaction strength and number between each cell type in high ISRS cells and low ISRS cells. **D** Chord chart and bubble chart showed overexpressed ligand–receptor interactions in high ISRS cells. Bubble size represents *P* value generated by the permutation test, and the color represents the possibility of interactions. **E** Heatmap showed the efferent or afferent contributions of all signals to different cell types in low ISRS cells (left) and low ISRS cells (right). **F** Dot plot shows dominant senders and receivers in high ISRS cells and low ISRS cells. The *X* and *Y* axes are the total outgoing or incoming communication probabilities associated with each cell group, respectively. The size of the dots is positively related to the number of inferred links (both outgoing and incoming) associated with each cell block. The colors of the dots represent different cell groups. **G** SCENIC analysis indicated significant regulons in low ISRS cells (left) and high ISRS cells (right)

To comprehensively verify the efficacy of ISRS in single-cell RNA-sequencing data, we utilized inferCNV algorithm to investigate the clonal structure of NE cells, endothelial cells, fibroblasts and Schwann cells. The infer CNV analysis revealed that NEs with chromosome 17q gain could serve as malignant cells, while cells with more CNVs owned higher ISRS scores in 4 NB single-cell datasets, validating the capability of risk stratification of ISRS (Fig. 10A, B, Supplementary Figure S7A–C). In cell communication analysis, we plotted circle diagrams to demonstrate the interaction times and interaction strengths among each cell type, visualizing the integrated cell communication networks in high ISRS cells and low ISRS cells. We compared the cell communication patterns between two ISRS groups, which revealed that B cells, myeloid cells, Schwann cells and fibroblasts might contribute most to differences in cell communication networks (Fig. 10C). Hence, we investigated over-expressed ligand-receptor pairs and their interactions among B cells, myeloid cells, Schwann cells and fibroblasts in the high ISRS group, identifying differential cell interactions between two ISRS groups (Fig. 10D). Different cell types would emit different contributive signals on the overall, incoming and outgoing signals between two ISRS groups, especially for Schwann cells, fibroblasts and endothelial cells (Fig. 10E, F, Supplementary Figure S7D). Besides we further investigated the relation between regulon (TFs and target genes) activity and each cell type in different ISRS group based on SCENIC analysis, which indicated that regulons of SOX4 and SMAD5 scored high activity in high ISRS cells, and regulons of KLF7 and SMARCA4 scored high activity in low ISRS cells (Fig. 10G, Supplementary Figure S7E).

### Pan-cancer analysis and immunohistochemistry of two hub genes

**Fig. 11:**
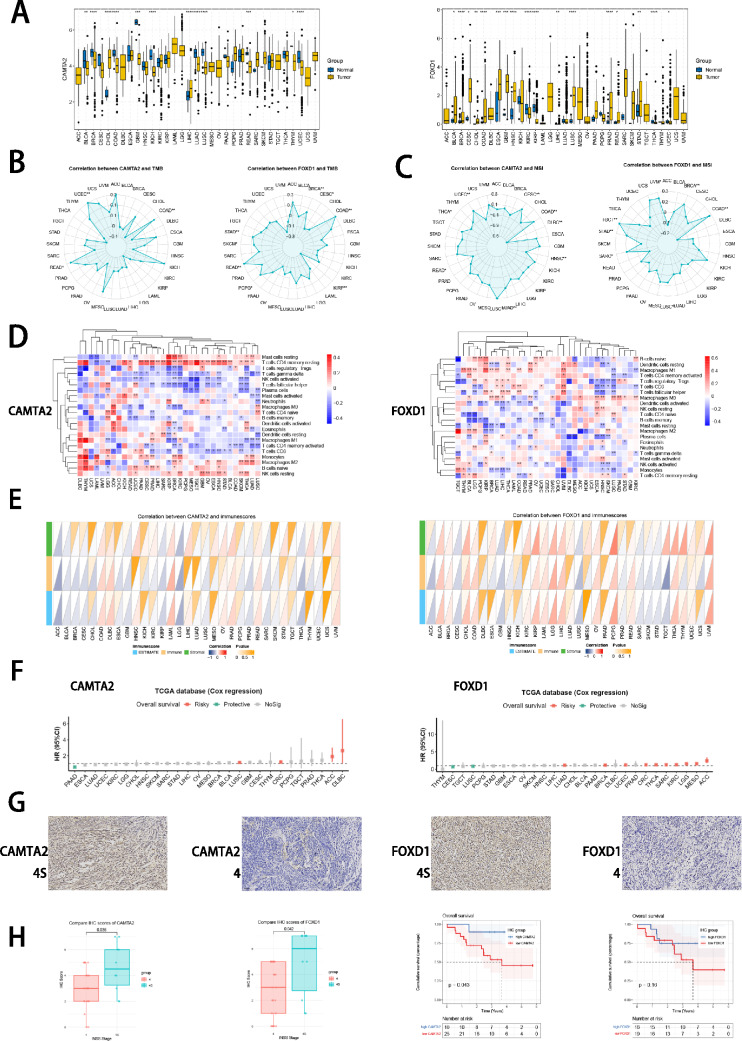
Pan-cancer analysis and experimental validation of two hub genes (CAMTA2 and FOXD1). **A** Differential expressions of CAMTA2 and FOXD1 in tumor and normal samples across 33 tumor types. **B**, **C** Correlation analysis between expressions of hub genes and TMB/MSI scores. **D**, **E** Correlation analysis between expressions of hub genes and immune cell proportions/immune scores calculated by ESTIMATE. **F** Cox regression analysis of CAMTAA2 and FOXD1 in multiple tumor types. **G**, **H** Protein expression levels of CAMTA2 and FOXD1 were assessed by IHC in stage 4S tissues and stage 4 tissues. Survival analysis was employed to validate the prognostic value of protein expressions of CAMTA2 and FOXD1

Finally, we observed two ISRS model genes (CAMTA2 and FOXD1) with abundant research value in oncology, which have been proven as oncogenes in head and neck squamous cell carcinoma (Wu et al. 2023) or colon cancer (Luan et al. 2021). Hence, we performed pan-cancer analysis to reveal the heterogeneity of CAMTA2 and FOXD1 expressions in tumor and normal samples across 33 tumor types (Fig. 11A). Meanwhile, correlation between expressions of two hub genes and TMB, MSI, immune cell and immune score highlighted the significance of two hub genes involved in tumor immune microenvironment and immune cell infiltration (Fig. 11B–E). The protective prognosis value of CAMTA2 was observed in PAAD, while that of FOXD1 was found in CESC and LUSC, showing a similar prognostic effect in NB (Fig. 11F). Furthermore, we conducted IHC staining to validate the significantly higher protein level of CAMTA2 and FOXD1 expression in stage 4S tissues than in stage 4 tissues, which supported our bioinformatics results (Fig. 11G, H, Supplementary Figure S7F). Meanwhile, we divided 35 stage 4S and 4 NB patients into high and low CAMTA2/FOXD1 group based on the median IHC score of CAMTA2/FOXD1. Survival analysis revealed that NB patients with high CAMTA2 protein levels had significantly better OS than NB patients with low CAMTA2 protein levels, while this difference was also observed between high and low FOXD1 groups, without significance (Fig. 11H).

## Discussion

Neuroblastoma (NB), most commonly seen in children under five years old, is a major life-threatening condition that contributes to roughly 15% of pediatric tumor-related deaths (Gurney et al. 1995). Varied clinical symptoms and molecular features of NB complicate the diagnosis and treatment efforts for clinicians (Aygun 2018). In advanced stages, complete surgical removal of primary tumors is often not possible, largely due to the tumor encircling and eroding the neurovascular system after extensive growth. Additionally, advanced stages are frequently accompanied by conditions such as distant metastasis and drug resistance, leading to a particularly poor prognosis for NB patients (Bhatnagar and Sarin 2012). This challenging context has brought the phenomenon of spontaneous regression, especially prevalent in stage 4S, into sharp focus among researchers. However, this phenomenon is not unique to stage 4S NB, while some NB children with bone metastases and low stages also have spontaneous regression (Matthay 1998). In addition, researchers also find that stage 4S may sometimes progress to stage 4 or other high-risk diseases, and even regress after progression (Tas et al. 2020). Yet, the mechanisms of spontaneous regression in NB remain elusive.

However, with the development of bioinformatics and multi-omics, we are striving to improve our understanding and management of this complex disease. For example, proteomics-based analysis demonstrated that some proteins, which were related to differentiation, proliferation and apoptosis, expressed differently between stage 4 and 4S NB with significance (Yu et al. 2011). Epigenetic analysis revealed that changes in gene expression related to promoter methylation, histone modification, or chromatin remodeling may affect the differentiation of NB (Das et al. 2013). Hence, we performed multiple methods of bioinformatics analysis to identify differentially methylated genes between INSS 4 and 4S stage NB, as well as developed an ISRS model to accurately diagnose stage 4 NB and forecast the prognosis of NB patients, which also showed a well capability to predict immunotherapy response and to risk-stratify NB patients in several aspects, such as immune microenvironment, mutation landscape, chemotherapy response and single-cell level evidences. With experimental validation of two differential expressed genes between INSS 4 and 4S stage NB, our research findings would offer valuable insights in deciphering tumor microenvironment, shedding light on the biological underpinnings of both the pathogenesis and spontaneous regression of NB.

In our study, we especially focused on enhancers, which are DNA sequences in the genome ranging from 50 to 1500 bp in length and can bind to TF to promote the transcription of target genes. As critical regulatory components of DNA, enhancers are involved in numerous intricate regulatory networks that influence cancer-related genes. Mutations within tumors often result in the dysregulation of these enhancers, leading to the abnormal expression of genes involved in growth and development (Adhikary et al. 2021; Zhang et al. 2018). Therefore, ELMER analysis was conducted to identify differential methylated enhancers between stage 4 and 4S NB. Motif FOXK1_HUMAN.H11MO.0.A. was found significantly differentially expressed, which throws light on underlying enhancer-associated mechanisms between stage 4 and 4S NB, thus laying the foundation for future research on spontaneous regression of NB. Moreover, ML is a crucial method in our study, leveraging advanced algorithms to handle large, heterogeneous datasets automatically, particularly excelling in prediction tasks by identifying meaningful patterns (Goecks et al. 2020). Using the expression profiles of differentially methylated genes, we utilized an integrative ML pipeline to construct a consensus ISRS to diagnose stage 4 NB and predict the prognosis of NB. In total, 101 kinds of prognostic algorithms and 113 kinds of predictive algorithms were applied to the training cohort based on the LOOCV framework. Subsequent validations across four independent NB cohorts revealed that the most effective prognostic model was Enet (alpha = 0.6) and the most effective diagnostic model was RF. The strength of this integrated approach lies in its ability to amalgamate various ML algorithms to create models with consistent diagnostic or prognostic capabilities for NB, which reduces the dimensionality of multiple variables to simplify the model for practical and translational use. The ISRS's efficacy as a prognostic tool was further underscored by time-dependent ROC curves, AUC values, calibration curves, and DCA curves, all of which highlighted its superiority over other clinical variables. Moreover, the meta-analysis of model performance in C-index demonstrated that ISRS maintained well accuracy and stability in external public cohorts, which suggested huge potential for its clinical application.

With the capacity of ISRS to categorize NB patients into high-risk and low-risk group, we were able to explore molecular differences and pathogenic mechanism between the two risk groups divided by ISRS model genes. Patients in the high-risk and low-risk group displayed huge biological distinctness in terms of immune microenvironment, immunotherapy response, somatic mutations and chemotherapy sensitivity. Based on DEGs between two risk groups and the model genes that consisted of ISRS, we performed functional enrichment analysis to reveal that the differential functions were primarily enriched in organ development, tissue morphogenesis and cell cycle. NB may be caused by abnormal differentiation of neural crest stem cells, which is associated with abnormal regulation of development, morphogenesis and cell cycle. The migration pathway of neural crest stem cells is the same as the tumor location of stage 4S NB, including the adrenal gland, liver, skin, and a small amount of bone marrow. Currently, most malignant tumors were found to contain stem cells or precursor-like cells with stem cell characteristics, which are associated with the influences of genetic and epigenetic changes on developing cells and mature cells (Ratner et al. 2016). Our functional enrichment analysis suggested that model genes of ISRS could be involved in the differentiation and maturation of NB cells, which might influence the differentiation related signals and pathways.

Nowadays, scRNA-seq technology is increasingly utilized in bioinformatics to explore the cellular components and interactions within the tumor microenvironment, which aids in identifying associations between tumor patterns and clinical outcomes and in assessing cell-specific drug effects for personalized cancer treatment (Hsieh et al. 2023). Besides, scRNA-seq studies have provided insights into the developmental origins of NB, which indicates that the degree of differentiation correlates with clinical prognosis, highlighting the importance of developmental biology in understanding and treating NB (Jansky et al. 2021). In our study, we performed single-cell analysis in four NB scRNA-seq datasets to further investigate the underlying cellular mechanisms of model genes that consisted of ISRS. Based on six algorithms of single-cell scoring, the expression profiles of model genes in ISRS were clearly visualized in single-cell levels, showing an abundance infiltration in NE cells and T cells. Subsequently, we performed pseudotime analysis and inferCNV algorithm in high ISRS cell and low ISRS cells, respectively, discovering significant differences in cellular differentiation and mutation landscape between the two ISRS groups. Cells with higher ISRS scores tended to be immature cells and malignant cells, which verified the capability of risk stratification of ISRS. Moreover, cell communication networks and transcription regulon networks in two ISRS revealed some differences, throwing light on a better understanding of tumor microenvironment and tumor heterogeneity.

Sequencing the genome or transcriptome of NB patient samples, collected through biopsy or surgical removal, could be a routine practice for diagnosis and guiding therapeutic decision-making. With sequencing data entered in ISRS, clinicians could precisely diagnose INSS stage 4 NB and predict the prognosis of NB patients, and tailor personalize treatment plan for patients with distant metastasis and poor prognosis. Meanwhile, ISRS can be easily duplicated via a PCR-based detection method, making it practicable in clinical transformation and implementation. However, we should admit certain limitations in our research. Firstly, our analysis was retrospective, with sequencing data and corresponding clinical data sourced from public databases, which needs a large-scale, multi-center prospective validation. The absence of therapy regimens, metastasis states and recurrence records might potentially distort our discoveries. Secondly, since the characteristics of CAMTA2 and FOXD1 in NB remains unclear, more real-world researches enrolling more tumor specimens, as well as more experiments in vitro or in vivo should be carried out to explore their biological function in NB. Last but not least, our current approach, which relies solely on transcriptome and methylation sequencing, could be significantly enhanced through the integration of multi-omics data. For example, proteomics offers a closer view of the functional molecules that drive cancer progression and response to therapy, potentially uncovering novel biomarkers and therapeutic targets. Incorporating proteomic analysis could significantly enrich our understanding of the spontaneous regression of NB by providing direct insights into protein expression levels and post-translational modifications between INSS 4 and 4S NB, which are pivotal for cellular function and phenotype. The comprehensive integration analysis facilitates a deeper deciphering of biological processes and physiological mechanisms, thereby enhancing the stability and accuracy of predictive algorithms. Moreover, multi-omics integration brings a wealth of features to the analytical process, which are crucial for more nuanced learning algorithms. Deep learning, a sophisticated subset of ML, has the distinct advantage of autonomously identifying features critical for classification. This contrasts with traditional ML methods, where such features must be manually selected and inputted. Therefore, the development and application of new deep learning algorithms, coupled with the rich insights from integrating multi-omics data, present a promising strategy for advancing individualized medicine in NB patients. In the future, with various clinicopathological parameters, imaging data and multi-omics data integrated into a novel multimodal model, we believe that the artificial intelligence model could strongly assist the existing NB treatment protocols in clinical practice, after large-scale validation implemented in multi-center cohorts.

## Conclusion

For the first time, we successfully developed INSS stage-related signatures to precisely diagnose stage 4 NB and predict the prognosis of NB patients based on abundant machine learning algorithms and multi-omics data. After serious validations in model performances, immune microenvironment, somatic mutations, immunotherapy and chemotherapy, we proved that our signature exhibited stability and strength as a promising predictive biomarker and therapeutic target in NB. Meanwhile, enhancer genes CAMTA2 and FOXD1 were identified from the signature, which expressed significantly higher in stage 4S than in stage 4, providing potential investigation values in the field of differentiation and spontaneous regression in NB.

## Supplementary Information

Below is the link to the electronic supplementary material.

Supplementary file1 (DOCX 4262 KB)

Supplementary file2 (XLSX 6200 KB)

## Data Availability

Publicly available datasets were analyzed in this study. The names of the repositories and accession numbers can be found within the article/Supplementary Materials. Further inquiries can be directed to the corresponding author (Dawei He).
