# Supplementary material for: Integrative analysis with machine learning identifies diagnostic and prognostic signatures in neuroblastoma based on differentially DNA methylated enhancers between INSS stage 4 and 4S neuroblastoma
Source: J Cancer Res Clin Oncol. 2024 Mar 21;150(3):148. doi: 10.1007/s00432-024-05650-4 (PMC10957705; doi:10.1007/s00432-024-05650-4)

**Integrative analysis with machine learning identifies diagnostic and prognostic signatures in neuroblastoma based on differentially DNA methylated enhancers between INSS stage 4 and 4S neuroblastoma**

Authors: Shan Li 1,2,3, Tao Mi 1,2,3, Liming Jin 1,2,3, Yimeng Liu 1,2,3, Zhaoxia Zhang 1,2,3, Jinkui Wang 1,2,3, Xin Wu 1,2,3, Chunnian Ren 1,2,3, Zhaoying Wang 1,2,3, Xiangpan Kong 1,2,3, Jiayan Liu 1,2,3, Junyi Luo 1,2,3, Dawei He 1,2,3

Corresponding author: Dawei He 1,2,3

E-mail: hedawei@hospital.cqmu.edu.cn

Postal address: Zhongshan 2nd Road, No.136, Children’s Hospital of Chongqing Medical University, Yuzhong District, Chongqing, China

1 Department of Urology, Children's Hospital of Chongqing Medical University, Chongqing 400014, China.

2 Chongqing Key Laboratory of Children Urogenital Development and Tissue Engineering, Chongqing 400014, China.

3 China International Science and Technology Cooperation base of Child Development and Critical Disorders, National Clinical Research Center for Child Health and Disorders, Ministry of Education Key Laboratory of Child Development and Disorders, Chongqing Key Laboratory of Pediatrics, Children's Hospital of Chongqing Medical University, Chongqing 400014, China.

SUPPLEMENTARY FIGURE 1

Batch effect removal, model evaluation and feature selection of ISRS. (A-B) PCA plots and heatmap visualization of superior effectiveness of batch effect removal before (A) and after (B) batch effect removal, respectively. (C-D) AUC, PRAUC, accuracy, sensitivity, specificity, precision, cross-entropy, Brier scores and precision-recall curve of top five ML combination in diagnostic ISRS. (E-F) The number of trees determined by minimum error and the importance of 67 model genes based on RF algorithm. (G) Logarithmic loss, recall and decision calibration of top five ML combination in prognostic ISRS. (H-I) Cvfit and lambda curves of elastic network applied with minimum lambda criteria. (J) The feature importance visualization of 20 variables selected by Enet demonstrated that CAMTA1 impacted most in the Enet model. (K) The flowchart to schematically explain the algorithmic pipeline of machine learning algorithm integration.


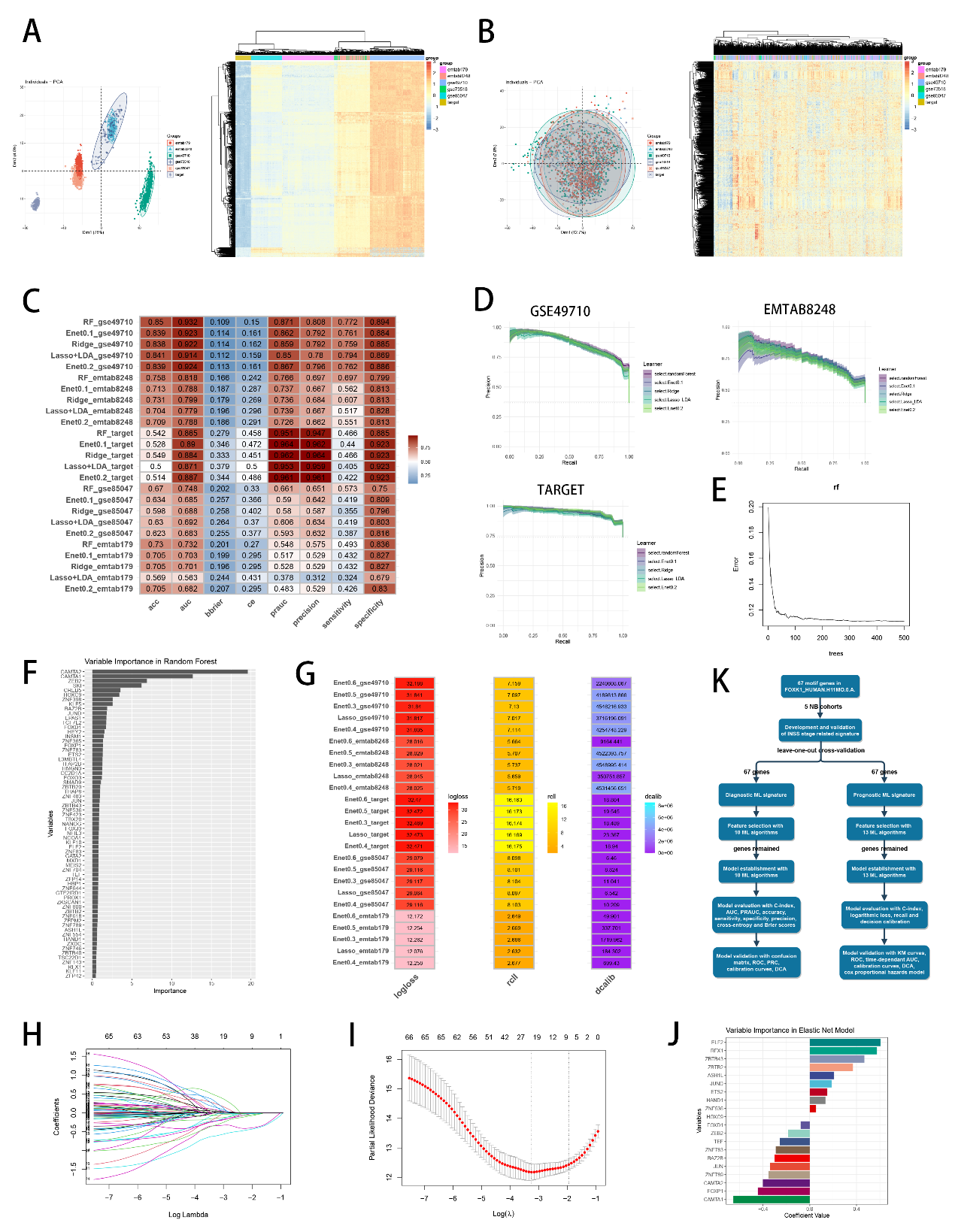


SUPPLEMENTARY FIGURE 2

Model validation of diagnostic ISRS based on machine learning algorithms. (A) Confusion matrix plotted in training (GSE49710) and validation cohorts (TARGET, GSE85047 and E-MTAB179) showed well accuracy of ISRS. (B) ROC curves and AUC in training (GSE49710) and validation cohorts (TARGET, GSE85047 and E-MTAB179) showed well discrimination of ISRS. (C) Nomogram visualized the logistic regression model based on ISRS and clinical variables. (D) DCA curves in training (GSE49710) and validation cohorts (TARGET, GSE85047 and E-MTAB179) showed well clinic benefit of ISRS.


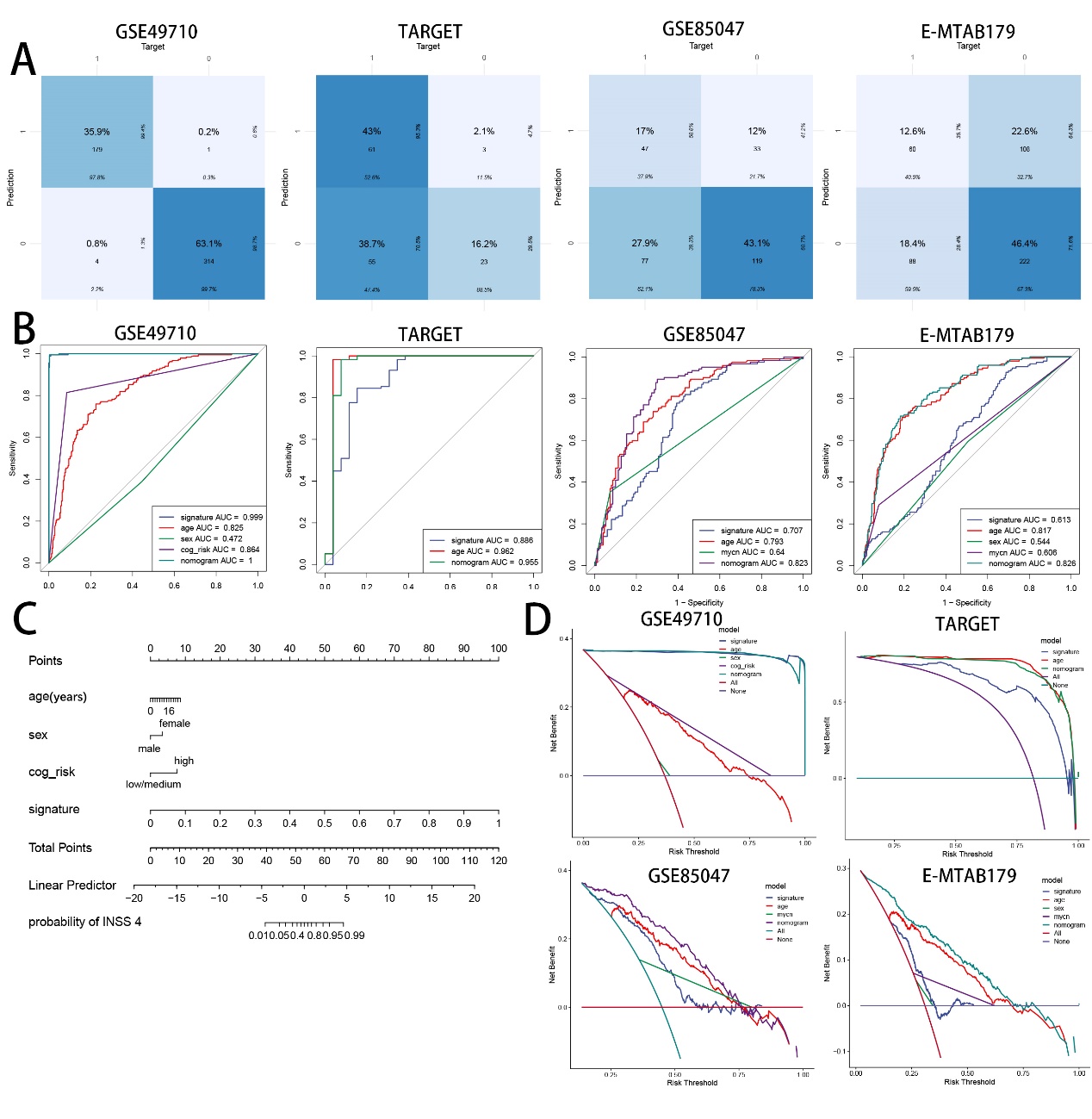


SUPPLEMENTARY FIGURE 3

Model validation of prognostic ISRS based on machine learning algorithms. (A) KM curves verified the discrimination ability of the ISRS signature to divide high-risk and low-risk patients. In the testing cohort E-MTAB 8248, TARGET and E-MTAB 179, the low-risk group owned a relatively longer OS and EFS than the high-risk group. In the testing cohort GSE85047, the low-risk group owned a relatively longer OS and PFS than the high-risk group. (B) ROC curves of 1-, 3- and 5- year OS in 4 validation cohorts showed well discrimination of ISRS signature. (C) ROC curves of 3- year OS in 4 validation cohorts (E-MTAB 8248, TARGET, GSE85047 and E-MTAB 179) showed that ISRS signature and cox regression model outperformed other clinical variables. (D) Nomogram visualized the cox regression model based on ISRS and several clinical variables. (E) Time dependent ROC curves in 4 validation cohorts (E-MTAB 8248, TARGET, GSE85047 and E-MTAB 179) indicated that ISRS and cox regression model outperformed conventional clinical variables in capability of discrimination. (F) Calibration curves in 4 validation cohorts (E-MTAB 8248, TARGET, GSE85047 and E-MTAB 179) showed well consistency of ISRS. (G) DCA curves in 4 validation cohorts (E-MTAB 8248, TARGET, GSE85047 and E-MTAB 179) showed well clinical benefit of ISRS. (H) Multivariate Cox regression analysis in 4 validation cohorts (E-MTAB 8248, TARGET, GSE85047 and E-MTAB 179) showed that ISRS could serve as an independent prognostic factor for NB patients (P < 0.05). Risk: risk scores calculated by prognostic ISRS.


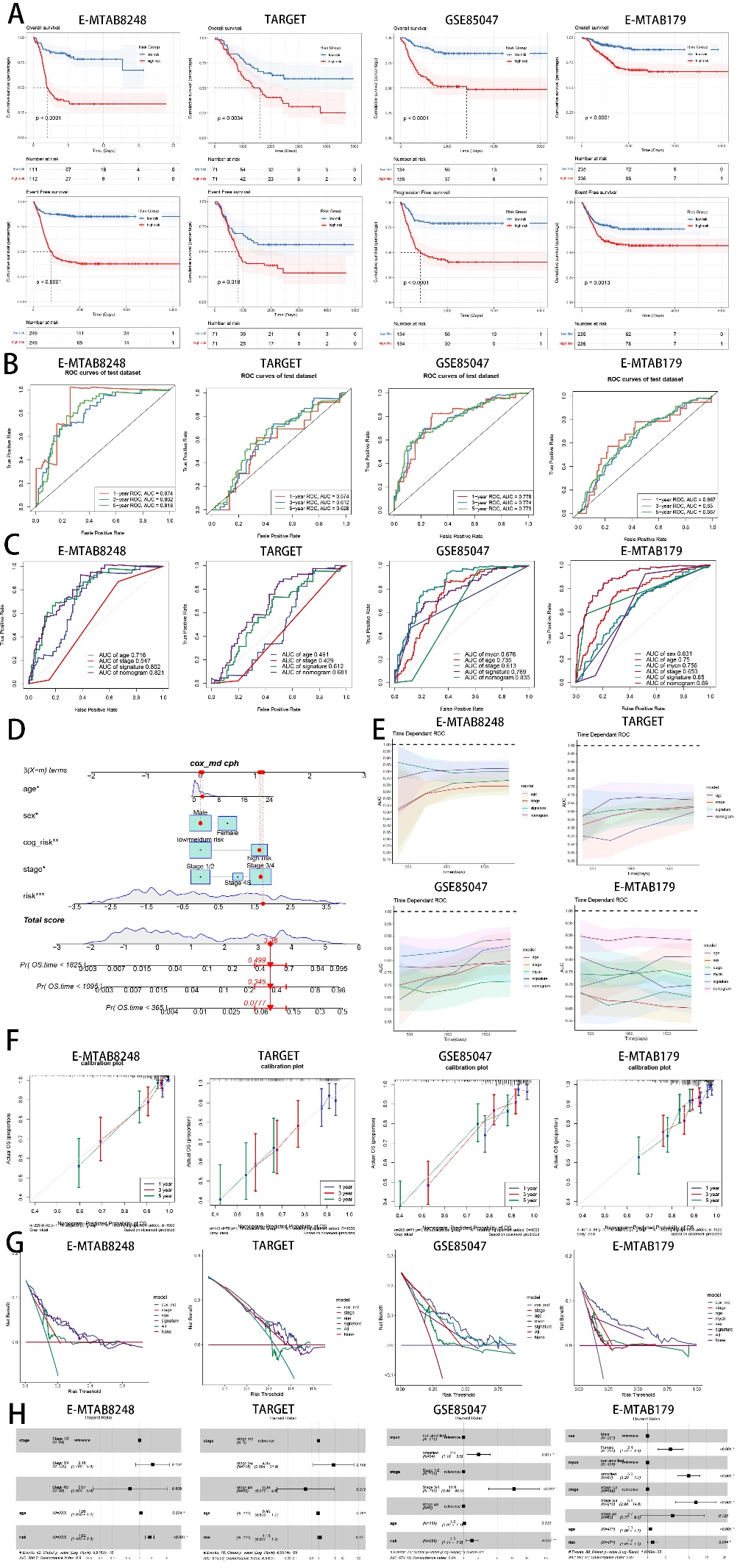


SUPPLEMENTARY FIGURE 4

Landscape of six ISRS model genes (ZEB2, CAMTA1, BAZ2B, CAMTA2, FOXD1 and HOXC9). (A) Boxplot visualized the significant differences in gene expressions between stage 4 and 4S. (B) K-M analysis revealed significantly protectively prognostic values of six model genes.


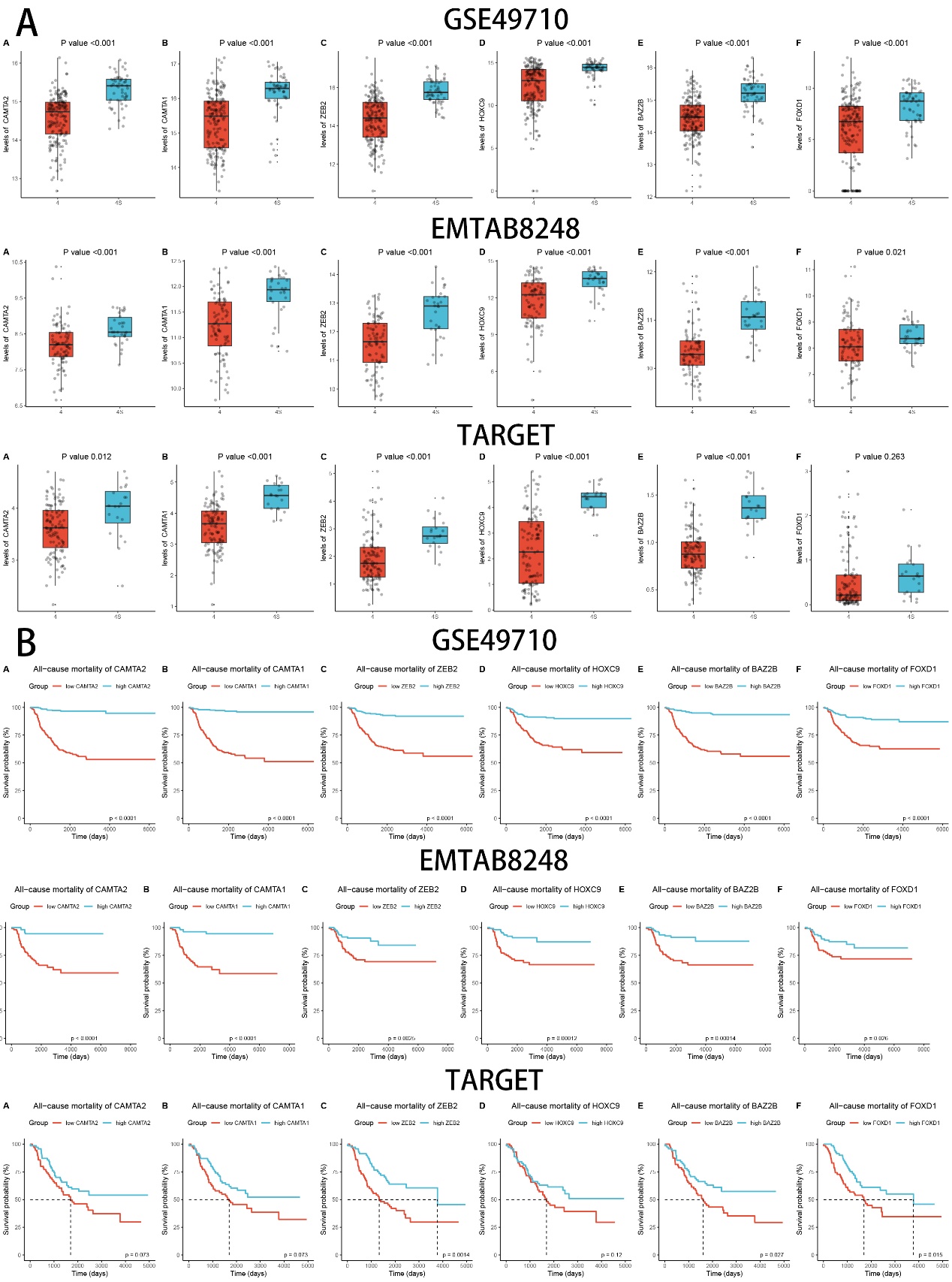


SUPPLEMENTARY FIGURE 5

Identification of ISRS model genes related clusters. (A) Consensus matrixes of NB patients for k = 2 in three NB cohorts (GSE49710, E-MTAB 8248, TARGET). (B) Cumulative distribution function curves for unsupervised clustering of NB patients for each k (k = 2–7) in three NB cohorts (GSE49710, E-MTAB 8248, TARGET). (C) tSNE analysis of two clusters. (D-E) GSEA and GSVA analysis indicated significant enrichment of pathways in cluster 1. (F-G) Visual summary displayed common genetic alterations in the cluster 1 and cluster 2. (H) Tumor mutation burdens between two clusters. (I-J) Comprehensive survival analysis on OS and EFS based on two clusters and two TMB groups.


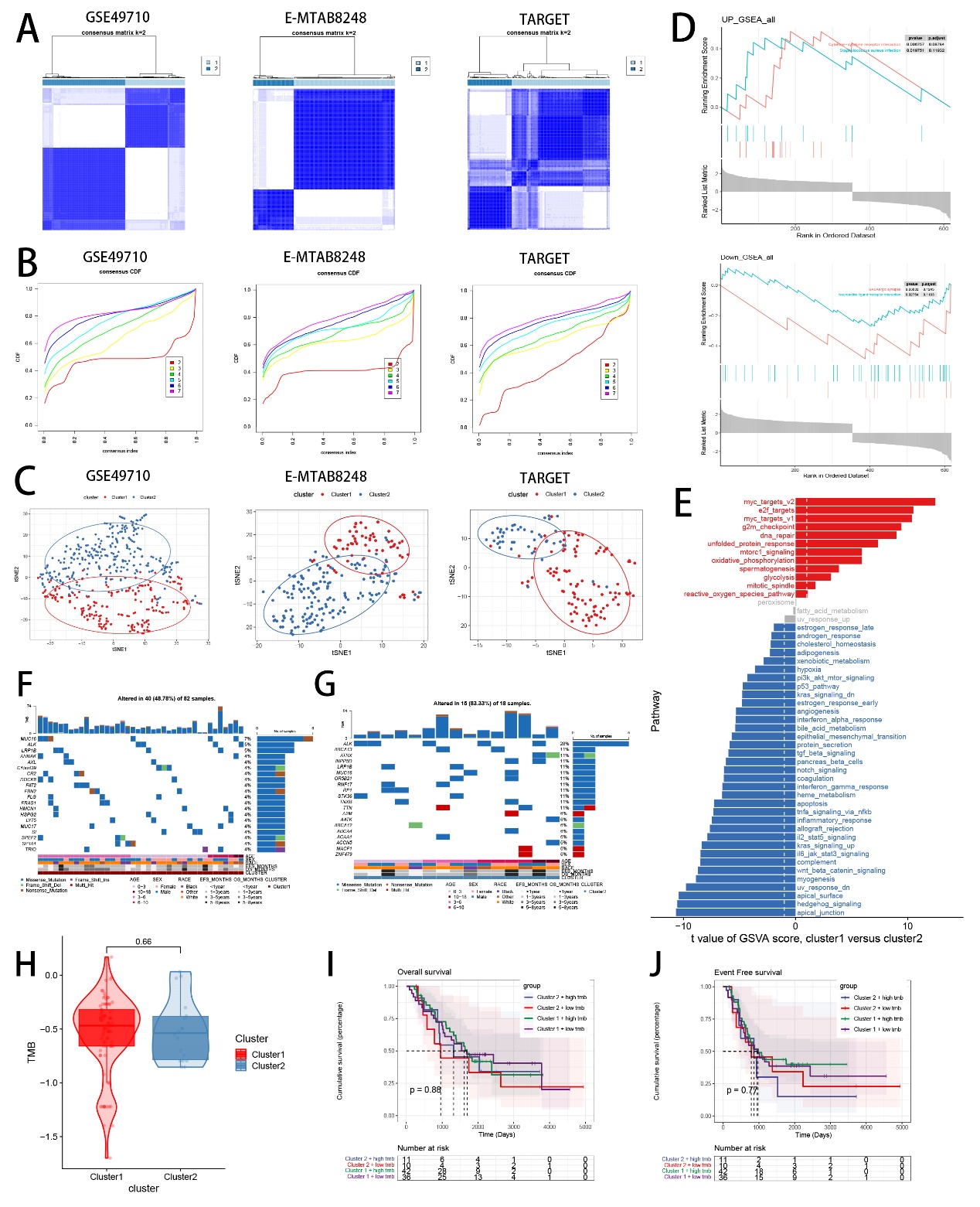


SUPPLEMENTARY FIGURE 6

Validation of ISRS and model genes in four scRNA-seq datasets. (A-B) Single cell scoring results of ISRS model genes based on another five algorithms (PercentageFeatureSet, AddModuleScore, AUCell, ssGSEA and Ucell) in each cell type in GSE137804. (C) UMAP plot visualized the cell annotation results and FeaturePlot visualized the expression profiles of model genes in three scRNA-seq datasets (GSE192906, GSE140819 and CellAtlas). (D-I) Single cell scoring results of ISRS model genes based on six algorithms (PercentageFeatureSet (D), AddModuleScore (E), AUCell (F), ssGSEA (G), Ucell (H) and singscore (I)) in each cell type. Left: CellAtlas; Middle: GSE140819; Right: GSE192906.


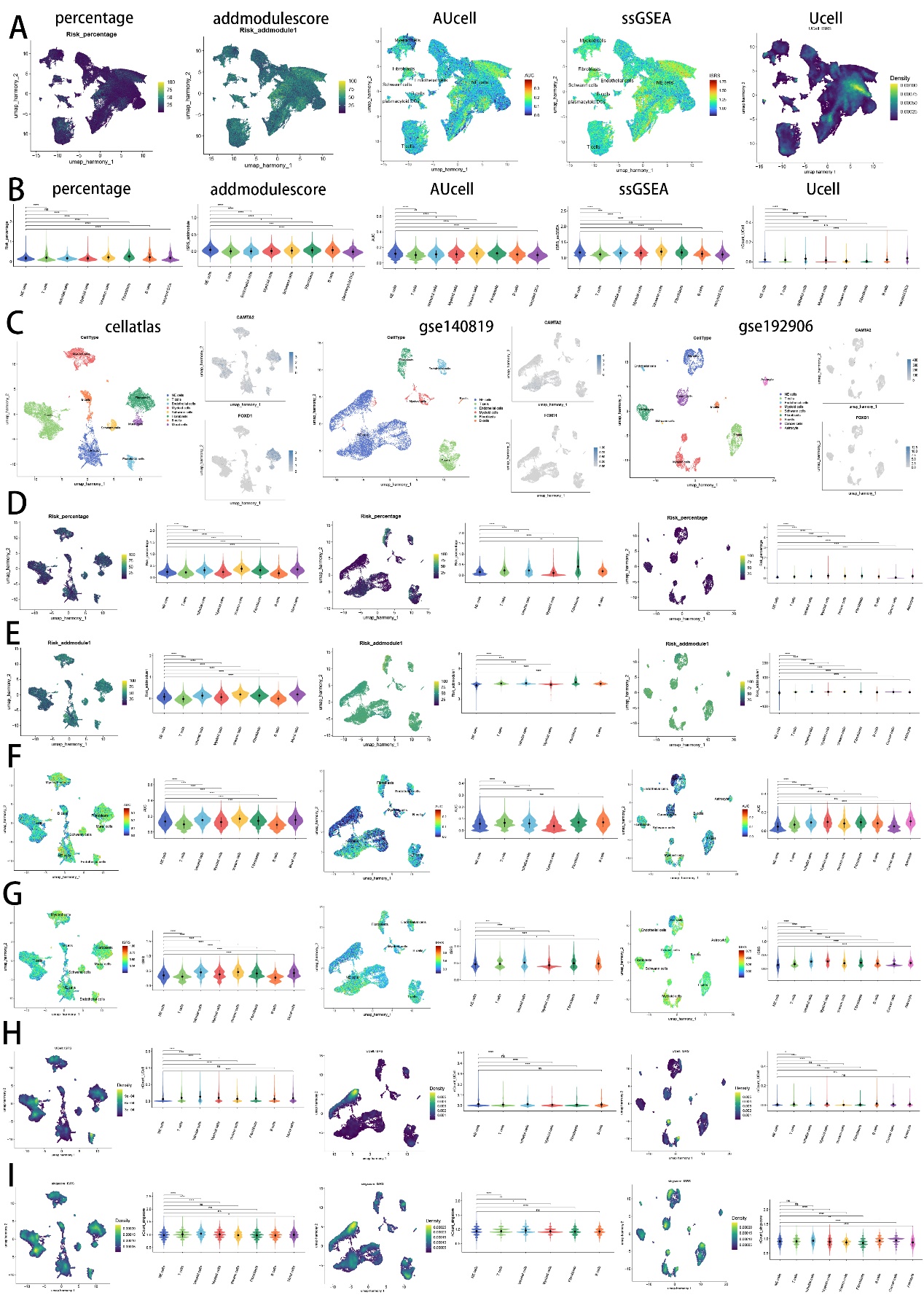


SUPPLEMENTARY FIGURE 7

The landscape of CNV, cell-cell communication, transcriptional regulons, and validation of model genes in experiments. (A-C) Differences of CNVs of NE cells, fibroblasts, Schwann cells and endothelial cells in high ISRS cells and low ISRS cells in GSE192906, GSE140819 and CellAtlas cohorts. (D) Heatmap showed the efferent or afferent contributions of all signals to different cell types in high ISRS cells and low ISRS cells. (E) SCENIC analysis ranked significant regulons of each cell type in high ISRS cells and low ISRS cells. (H) Protein expression levels of CAMTA2 and FOXD1 were assessed by IHC in stage 4S tissues and stage 4 tissues.


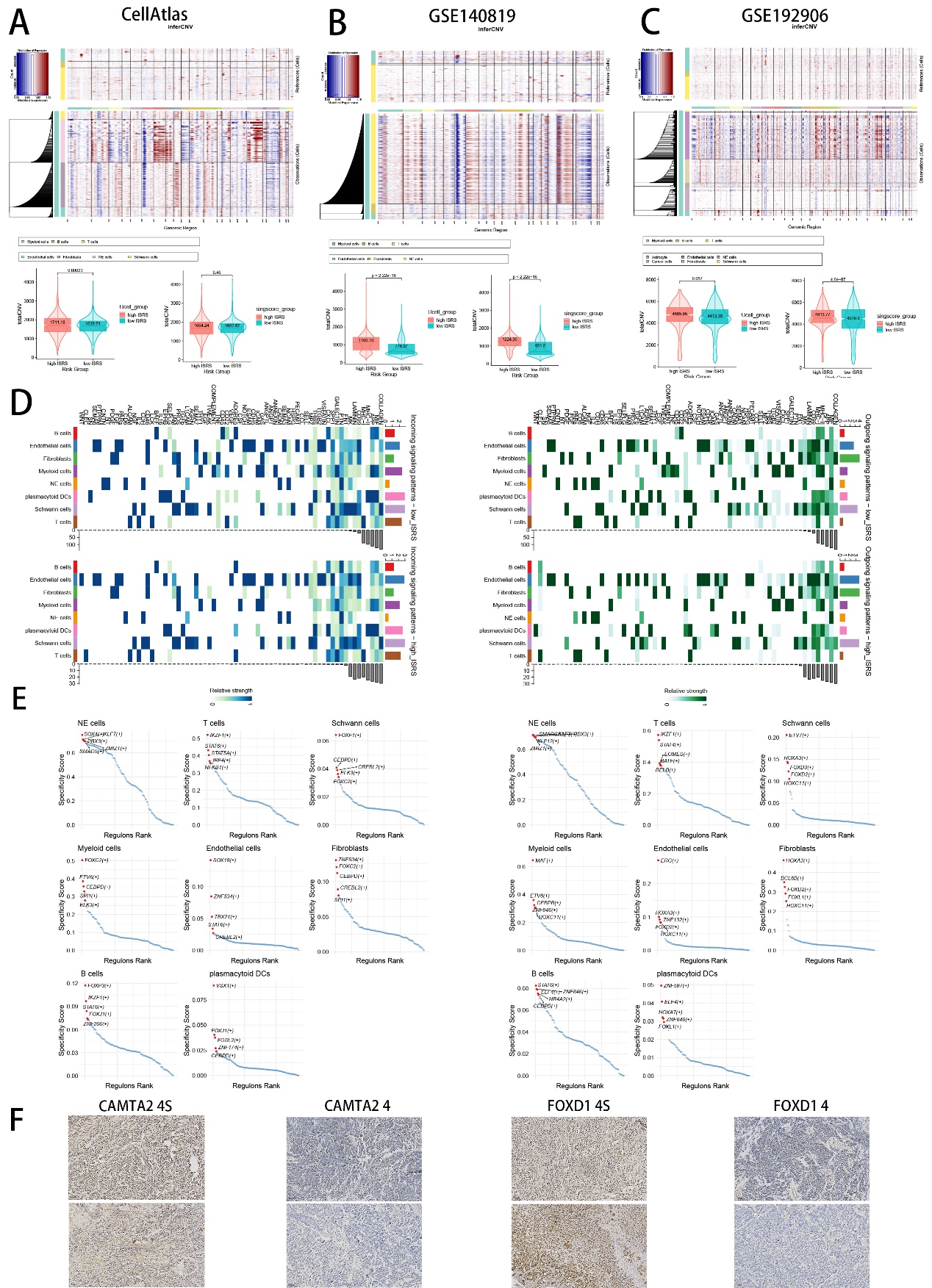

Supplement: Supplementary file 1 — Supplementary file1 (DOCX 4262 KB) [file 432_2024_5650_MOESM1_ESM.docx]
